# Expression Patterns of Genes Involved in Sugar Metabolism and Accumulation during Apple Fruit Development

**DOI:** 10.1371/journal.pone.0033055

**Published:** 2012-03-07

**Authors:** Mingjun Li, Fengjuan Feng, Lailiang Cheng

**Affiliations:** 1 College of Horticulture, Northwest A&F University, Yangling, Shaanxi, China; 2 Department of Horticulture, Cornell University, Ithaca, New York, United States of America; Friedrich-Alexander-University Erlangen-Nurenberg, Germany

## Abstract

Both sorbitol and sucrose are imported into apple fruit from leaves. The metabolism of sorbitol and sucrose fuels fruit growth and development, and accumulation of sugars in fruit is central to the edible quality of apple. However, our understanding of the mechanisms controlling sugar metabolism and accumulation in apple remains quite limited. We identified members of various gene families encoding key enzymes or transporters involved in sugar metabolism and accumulation in apple fruit using homology searches and comparison of their expression patterns in different tissues, and analyzed the relationship of their transcripts with enzyme activities and sugar accumulation during fruit development. At the early stage of fruit development, the transcript levels of sorbitol dehydrogenase, cell wall invertase, neutral invertase, sucrose synthase, fructokinase and hexokinase are high, and the resulting high enzyme activities are responsible for the rapid utilization of the imported sorbitol and sucrose for fruit growth, with low levels of sugar accumulation. As the fruit continues to grow due to cell expansion, the transcript levels and activities of these enzymes are down-regulated, with concomitant accumulation of fructose and elevated transcript levels of tonoplast monosaccharide transporters (TMTs), *MdTMT1* and *MdTMT2*; the excess carbon is converted into starch. At the late stage of fruit development, sucrose accumulation is enhanced, consistent with the elevated expression of sucrose-phosphate synthase (SPS), *MdSPS5* and *MdSPS6*, and an increase in its total activity. Our data indicate that sugar metabolism and accumulation in apple fruit is developmentally regulated. This represents a comprehensive analysis of the genes involved in sugar metabolism and accumulation in apple, which will serve as a platform for further studies on the functions of these genes and subsequent manipulation of sugar metabolism and fruit quality traits related to carbohydrates.

## Introduction

Carbohydrates provide energy and building blocks for plant growth and development. In addition, soluble sugars, including sucrose (Suc), glucose (Glc) [1], [2] and fructose (Fru) [3], [4], are known to act as signal molecules to regulate the expression of many key genes involved in plant metabolic processes and defense responses, consequently regulating plant growth and development [1], [5]. Carbohydrates are also central to quality and yield of crops. In fleshy fruits, the accumulation of soluble sugars during fruit development largely determines their sweetness at harvest.

**Figure 1:**
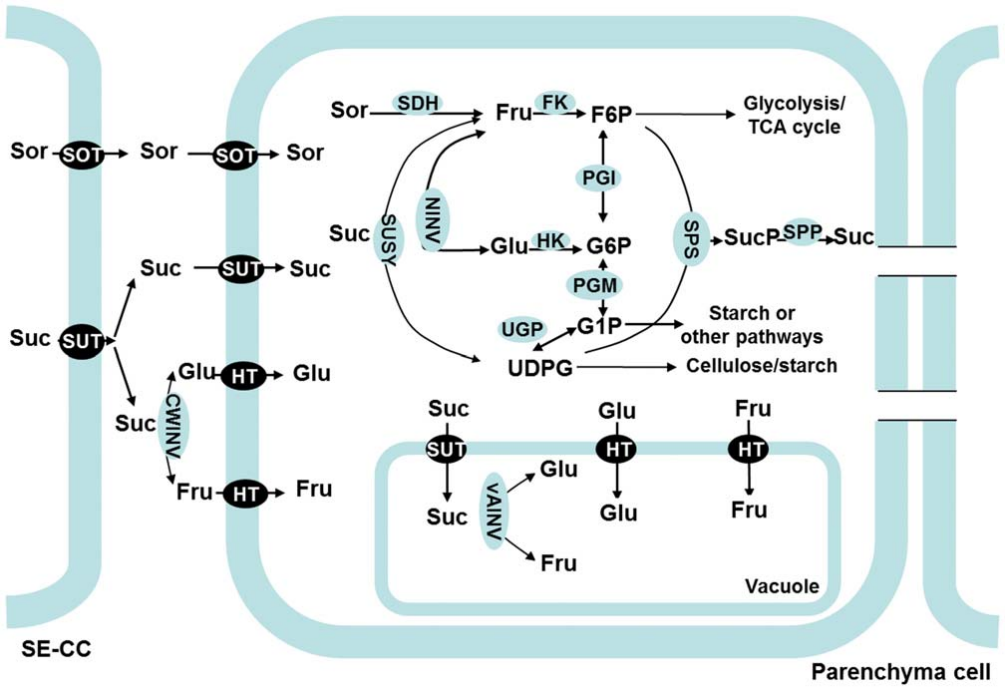
Sugar metabolism and accumulation in apple fruit [1], [14]. Both sorbitol (Sor) and sucrose (Suc) are unloaded to the cell wall space between sieve element-companion cell complex (SE-CC) and parenchyma cells in fruit [14]. Sor is taken up into parenchyma cells via sorbitol transporter (SOT). Suc is directly transported into parenchyma cells by plasma membrane-bound sucrose transporter (SUT), or converted to fructose (Fru) and glucose (Glc) in the cell wall space by cell wall invertase (CWINV), and then transported into the parenchyma cells by hexose transporter (HT). In the cytosol, Sor is converted to Fru by sorbitol dehydrogenase (SDH), while Suc can be converted to Fru and Glc by neutral invertase (NINV) or to Fru and UDP-glucose by sucrose synthase (SUSY). The resulting Glc and Fru can be phosphorylated to glucose 6-phsophate (G6P) and fructose 6-phosphate (F6P) by hexokinase (HK) and fructokinase (FK, specific for Fru). The conversions between F6P, G6P, G1P, and UDPG are catalyzed by phosphoglucoisomerase (PGI), phosphoglucomutase (PGM), and UDPG-pyrophosphorylase (UGP) in readily reversible reactions. The F6P produced in sugar metabolism enters glycolysis/TCA cycle to generate energy and intermediates for other processes. G1P is used for starch synthesis. UDPG can be used for cellulose synthesis or combined with F6P for re-synthesis of Suc via sucrose phosphate synthase (SPS) and sucrose-phosphatase (SPP). Most of the Fru, Glc and Suc that have not been metabolized are transported by special tonoplast transporters into vacuole for storage. Inside the vacuole, Suc can be also converted to Glc and Fru by vacuolar acid invertase (vAINV).

Plants have evolved an elaborate system for sugar metabolism and accumulation in sink cells (Figure 1). Here, we name this system as Suc-Suc cycle (previously called ‘futile recycles’ [6]). In this system, once Suc is transported into sink cells (e.g. fruit, root or shoot tips), it is converted to Fru and Glc by neutral invertase (NINV, EC 3.2.1.26), or to Fru and UDP-glucose (UDPG) by sucrose synthase (SUSY, EC 2.4.1.13). The resulting Glc and Fru are then phosphorylated to glucose 6-phosphate (G6P) and fructose 6-phosphate (F6P) by hexokinase (HK, EC 2.7.1.1) and fructokinase (FK, EC 2.7.1.4). The interconversions between F6P, G6P, G1P, and UDPG are catalyzed by phosphoglucoisomerase (EC 5.3.1.9), phosphoglucomutase (EC 5.4.2.2) and UDP-glucose pyrophosphorylase (EC 2.7.7.9) in readily reversible reactions. The F6P produced in sugar metabolism enters glycolysis and the TCA cycle to generate energy and intermediates for other processes; G1P is used for starch synthesis; and both F6P and UDPG can be combined to re-synthesize Suc via sucrose phosphate synthase (SPS, EC 2.4.1.14) and sucrose-phosphate phosphatase (EC 3.1.3.24) [1]. Most of the Suc, Glc and Fru and other soluble sugars that have not been metabolized are transported into the vacuole by special transporter proteins located on the vacuole membrane. Once inside the vacuole, Suc can also be converted to Glc and Fru by vacuolar acid invertase (vAINV) [1]. This system operates in such a way that it not only allows carbon to be allocated into different pathways to satisfy sink growth and development, but also coordinates sugar metabolism and accumulation and maintains the balance in osmotic potential and turgor between cytosol and other subcellular compartments.

The concentration and distribution of sugars in plant cells are modulated by this Suc-Suc cycle system, which are affected by both internal and external factors, such as developmental processes [7]–[9], tissue types [10] and environmental conditions [5]. For example, rapid Suc accumulation in ripening fruit of melon (*Cucumis melo*) is attributed to higher SPS activity and lower invertase activity compared with young fruit, whereas activities of HK and FK are decreased [8]. In grape (*Vitis vinifera*) berries, the expression of two hexose transporter (HT) family genes, tonoplast monosaccharide transporter (TMT) and vacuolar glucose transporter (vGT) corresponds to massive accumulation of Glc and Flu in the vacuole [11]. The expression levels of hexokinases, *OsHK5* and *OsHK6*, which function as Glc sensors, are up-regulated in rice (*Oryza sativa*) leaves by exogenous application of Glc and Fru [12]. However, our current understanding of the mechanisms controlling the homeostasis and accumulation of sugars in fleshy fruit still remains quite limited.

Apple (*Malus domestica* Borkh), a member of the *Rosaceae* family, is among the most important commercial fruit crops grown worldwide. Apple and other *Rosaceae* tree fruits synthesize sorbitol (Sor), in addition to Suc, in source leaves, and both Sor and Suc are translocated to and utilized in fruit, with sorbitol accounting for about 60–70% of the photosynthates produced in leaves and transported in the phloem [13]. After being unloaded from SE-CC complexes into the cell wall space in apple fruit [14], Sor is taken up into the cytosol of parenchyma cells by sorbitol transporter (SOT), and then converted to Fru by sorbitol dehydrogenase (SDH, EC 1.1.1.14) [15] (Figure 1); Suc is directly transported into parenchyma cells by sucrose transporters (SUT or SUC) located on the plasma membrane, or converted to Glc and Fru by cell wall invertase (CWINV) first, and then transported into parenchyma cells by hexose transporters [14], [16]. Compared with sink organs in model plants that import and metabolize sucrose alone (e.g. *Arabidopsis*, *Solanum tuberosum*, and *Populus*), apple is unique in the metabolism and accumulation of sugars: more than 80% of the total carbon flux goes through Fru(because almost all the sorbitol is converted to Fru and half of the sucrose is converted to Fru), and Fru accumulates to a much higher level than Glc in the fruit. Although there have been some reports on the accumulation of carbohydrates [9], [13], [17] and changes in the activities of related enzymes during apple fruit development [13], [14], [17], it remains unclear how sugar metabolism and accumulation is regulated at the gene expression level.

In this article, we identified members of the various gene families that encode key enzymes or transporters involved in sugar metabolism and accumulation using homology analysis based on *Malus* genome [18] and EST sequences and comparison of expression patterns in different tissues, and analyzed the relationship of their relative transcript abundance and activities of enzymes with sugar accumulation during apple fruit development.

## Materials and Methods

### Plant materials

Five-year-old ‘Greensleeves’ apple (*M. domestica* Borkh.) trees on M. 26 rootstocks were used in this study. All the trees were grown in 55-L plastic containers in a medium of 1 sand: 2 MetroMix 360 (v/v) (Scotts, Marysville, OH, USA) outdoors under natural conditions in Ithaca, NY, USA. They were trained as a spindle system and grown at a density of 1.5×3.5 m. The cropload of these trees was adjusted by hand thinning to 4 fruit per cm^2^ trunk cross-sectional area at 10 mm king fruit size. They were supplied with 15 mM N using Plantex® NPK (20–10–20) with micronutrients (Plantex Corp., Ontario, Canada) twice weekly during the growing season. Fungicides and pesticides were sprayed at regular intervals throughout the growing season to protect the plants from diseases and insects. At 40 (near the end of cell division), 74 (early stage of cell expansion), 108 (late stage of cell expansion), and 134 days (maturity) after bloom (DAB) [7], fruit samples were taken from the south side of the tree canopy between noon and 2:00PM under full sun exposure. On each sampling date, five replicates of fruit samples, with at least six fruit in each replicate from three trees, were harvested. The sampled fruit were immediately weighed, cut into small pieces after removing the core, and frozen in liquid nitrogen on site (It took about 2 min from harvest to frozen). To compare the expression patterns of related genes in source and sink tissues, we also obtained mature leaves, shoot tips at 40 DAB from the trees. All the frozen samples were stored at −80°C until use.

### Identification of candidate genes

Candidate genes were identified by performing Blastp analysis against apple gene set (nucleic acid), in the *Malus* Genome Database from ‘Fondazione Edmund Mach Istituto Agrario San Michele All'Adige’, Italy (http://genomics.research.iasma.it/blast/blast.html) [18] using *A. thaliana* invertase, *SUSY*, *HK*, *SPS*, *SUT*, *TMT* and *vGT* sequences (obtained from The Arabidopsis Information Resource (http://arabidopsis.org/) as query (except for *FK* using *Lycopersicon esculentum* [19] as query), and an E-value of 1,00E-04 as threshold. The putative candidate gene sequences were retrieved from the *Malus* Genome Database: http://genomics.research.iasma.it/gb2/gbrowse/apple/. The corresponding sequences of candidate genes were then used for a BLAST search against the *Malus* EST database in the National Center for Biotechnology Information (http://www.ncbi.nlm.nih.gov/) to confirm that each predicted gene is expressed in *Malus* transcriptome while there is a high similarity EST sequence (score >300 bp, and identity >98%). Then, all ESTs sharing high similarity (>98%) with predicted genes were subjected to contig assembly (score >300 bp, and identity >98%; http://mobyle.pasteur.fr/cgi-bin/MobylePortal/). After similarity analysis between the predicted gene and its EST-constructed contig or EST, the divergent gene in splicing again underwent a Blastp analysis against all predictions in apple (nucleic acid) (http://genomics.research.iasma.it/blast/blast.html) using EST-constructed contig or EST sequence so that a concordant sequence with EST would be found in all predictions. Forty-one putative candidate genes involved in sugar metabolism in apple, including 3 CWINVs, 3 NINVs, 3 vAINVs (Table S1), 5 SUSYs (Table S2), 4 FKs (Table S3), 6 HKs (Table S4), 6 SPSs (Table S5), 5 SUTs, 5 TMTs and 2 vGTs (Table S6), were screened for expression analysis. Additionally, representative *MdSOTs* (*MdSOT1*, Genbank accession, AY237401, low Km; *MdSOT2*, AY237400, high Km [20]) and *MdSDHs* were also used for expression analysis. Although 17 predicted *SDH* homology genes were found in *Malus* genome [18], only *MdSDH1* to *MdSDH9* (*SDH1*, AY244806; *SDH2*, AY244807; *SDH3*, AY244809; *SDH4*, AY053504; *SDH5*, AY244811; *SDH7*, AY244813, *SDH8*; AY244812; *SDH9*, AY244810) had been systematically investigated as NAD-dependent sorbitol dehydrogenase [21], [22]. Since *MdSDH2* shares high similarity of cDNA sequence with *MdSDH3* to *MdSDH9* [18], a pair of universal primers was designed for *MdSDH2*-*SDH9* based on their conserved cDNA region.

### Sequence similarities and phylogeny analyses

Sequence similarities were determined by performing Clustal V multiple alignments using Lasergene software (DNASTAR, USA). Phylogenetic analysis of *Malus* and *A. thaliana* or *L. esculentum* (Only for FK) amino acid sequences was performed using maximum likelihood (http://www.phylogeny.fr) [23]. For this, amino acid alignments were performed using the MUSCLE program [24], and maximum likelihood trees with 100 bootstrap replicates were constructed with the PHYML program [25] and the JTT amino acid substitution model. Phylogenic tree was visualized using Treedyn program [26]. Additionally, the subcellular localizations of candidate genes were predicted using the TargetP software (http://www.cbs.dtu.dk/services/TargetP; [27]) and WoLF PSORT version of PSORT II (http://wolfpsort.org/; [28]).

### mRNA expression analysis

Quantitative reverse transcription-polymerase chain reaction (qRT-PCR) was used to analyze expression of the genes involved in sugar metabolism and accumulation (Tables S1, S2, S3, S4, S5, 6). Total RNA was extracted from samples by the modified CTAB method [29], and DNase was used to clean out DNA before reverse-transcription. After analysis of sequence similarities, gene-specific primers (Table S7) were designed, using Primer5 software. Primer specificity was determined by RT-PCR and Melt Curve analysis. qRT-PCR was performed with a iScript cDNA Synthesis Kit (Bio-Rad) according to the manufacturer's protocol. The amplified PCR products were quantified by an iQ5 Multicolor Real-Time PCR Detection System (Bio-Rad Laboratories, Hercules, CA, USA), with iQ SYBR Green Supermix kit (Bio-Rad). *Actin* (CN938023) transcripts were used to standardize the different gene cDNA samples throughout the test. For all samples, five tubes of total RNA were extracted from five replicates, respectively, and then mixed in a tube used for reverse-transcription. qRT-PCR experiments were done with 3 technical replicates. The data were analyzed using the ddCT method in iQ5 2.0 standard optical system analysis software.

### Assay of enzyme activities

SDH was extracted according to Park et al. [15] with some modifications. Each sample (0.50 g) was homogenized in 2 ml of 100 mM potassium phosphate (pH 7.8) buffer, containing 1 mM EDTA, 1 mM dithiothreitol (DTT), 1% BSA, 0.2% Triton X-100 and 1% (w/v) insoluble polyvinylpolypyrrolidone (PVPP). The homogenate was centrifuged at 16 000 *g* for 10 min at 2°C and 1 ml of the supernatant was desalted with a Sephadex G25 PD-10 column (Amersham BioSciences, Piscatway, NJ, USA) equilibrated with 125 mM Tris-HCl (pH 9.6). SDH activity was assayed in 1.0 ml reaction mixture containing 300 mM Sor, 1 mM NAD^+^, and 0.2 ml of the desalted extract in 100 mM Tris-HCl (pH 9.6), and NADH production was determined at 340 nm.

To extract CWINV, NINV, vAINV, SUSY, FK, HK, and SPS, 0.5 g sample was homogenized in 2 ml of 200 mM Hepes-KOH (pH 8.0) containing 5 mM MgCl_2_, 2 mM EDTA, 2.5 mM DTT, 2 mM Benzamidina, 0.1 mM leupeptin, 0,1% BSA, 2% glycerol, 1% Triton X-100) with 4% PVPP, similar to that used by Moscatello et al. [30]. The extract was centrifuged at 16 000 *g* for 20 min at 4°C, and immediately desalted in a Sephadex G25 PD-10 column, equilibrated with the extraction buffer at the concentration of 50 mM of Hepes-(KOH) (pH 7.4) but without Triton X-100 or DTT. For CWINV, the pellet was washed three times with the desalting buffer, and the protein on cell wall was solubilized by incubation in the extraction buffer with 1 M NaCl added at 4°C overnight. Then the extract was centrifuged and desalted as above. The desalted extract was used to assay soluble protein content and CWINV activity, while an aliquot of the desalted extract was boiled for 5 min to denature the enzyme as blank for each sample.

CWINV and vAINV were assayed for 60 min at 37°C, in an 200 µl assay mixture containing 100 mM phosphate-citrate buffer (pH 4.8), 100 mM sucrose, and 50 µl of the desalted extract or denatured extract (as blank). The assays were stopped by boiling for 3 min before adding 0.75 M Tris-HCl buffer (pH 8.5). The assay conditions for the NINV were the same except that the assay mixture contained Hepes-KOH (pH 7.2) as buffer. The amount of glucose produced from sucrose was determined by the enzyme-coupling method [10].

SUSY activity was determined according to Dancer et al. [31]. The enzyme extract (20 µl) was incubated at 27°C for 30 min in 100 µl final volume of assay medium containing 20 mM Hepes-KOH (pH 7.0), 100 mM sucrose and 4 mM UDP. The reaction was stopped by boiling in water for 3 min. Blanks contained the same assay mixture, but denatured extract was used. The UDPG content produced in the assay was measured spectrophotometrically following the reduction of NAD^+^ coupled to UDPG dehydrogenase activity in a reaction mixture (1.0 ml) containing 5 mM MgCl_2_, 2 mM NAD^+^ and 0.02 U UDPG dehydrogenase, and 100 µl of the reaction mixture for SUSY in 200 mM glycine (pH 8.9). The mixture was incubated at 27°C for 30 min. and NADH production was determined at 340 nm.

HK and FK activities were assayed by a continuous spectrophotometric assay as used by Renz and Stitt [32] with minor modifications. For HK, the assay mixture (0.5 mL) contained 50 mM Tris-HCl (pH 8.0), 4 mM MgCl_2_, 2.5 mM ATP, 0.33 mM NAD^+^, 1 U of G6P dehydrogenase, 1 mM glucose and 25 µl of the desalted extract. For FK, one unit of phosphoglucoisomerase was also added and 0.4 mM Fru was used instead of Glc.

SPS was measured in a two-step assay, following the procedure of Stitt et al. [33]. The reaction mixture (200 µl total volumes) contained 50 mM Hepes-KOH (pH 7.4), 4 mM MgCl_2_, 1 mM EDTA, 4 mM F6P, 20 mM G6P, 3 mM UDPG and 100 µl of sample. The reaction was carried out at 27°C for 30 min and stopped by boiling in water for 3 min. Blanks were run, for each assay, by adding denatured extracts. After centrifugation for 1 min at 12 000 *g*, 75 µl of the reaction mixture was used for UDP measurement in a spectrophotometric assay in a final volume of 1.0 ml containing 50 mM Hepes-KOH (pH 7.0), 5 mM MgCl_2_, 0.3 mM NADH, 0.8 mM phosphoenolpyruvate, 14 U of lactate dehydrogenase, and 4 U of pyruvate kinase (to start the reaction).

### Measurements of soluble sugars and starch

Soluble sugars and hexose phosphates were extracted and derivatized according to Wang et al. [34]. Briefly, 0.1 g sample was extracted in 1.4 ml 75% methanol with ribitol added as internal standard. After fractionation of non-polar metabolites into chloroform, 2 and 100 µl of the polar phase of each sample were taken and transferred into 2.0 ml Eppendorf vials for highly abundant metabolites (such as Sor, Suc, Glc, and Fru) and less abundant metabolites (such as G6P and F6P), respectively. They were dried under vacuum without heating and then derivatized with methoxyamine hydrochloride and N-methyl-N-trimethylsilyl-trifluoroacetamide sequentially [35]. After derivatization, metabolites were analyzed with an Agilent 7890A GC/5975C MS (Agilent Technology, Palo Alto, CA, USA) [34]. Metabolites were identified by comparing fragmentation patterns with those in a mass spectral library generated on our GC/MS system and an annotated quadrupole GC–MS spectral library downloaded from the Golm Metabolome Database (http://csbdb.mpimp-golm. mpg.de/csbdb/gmd/msri/gmd_msri.html) and quantified based on standard curves generated for each metabolite and internal standard.

The tissue residue after 75% methanol extraction for GC-MS analysis was re-extracted with 80% (v/v) ethanol at 80°C three times, and the pellet was retained for determination of starch. After digesting the residue with 30 U of amyloglucosidase (EC 3.2.1.3) at pH 4.5 overnight, starch was determined enzymatically as glucose equivalents [36].

## Results

### Candidate genes encoding key enzymes involved in sugar metabolism

**Figure 2:**
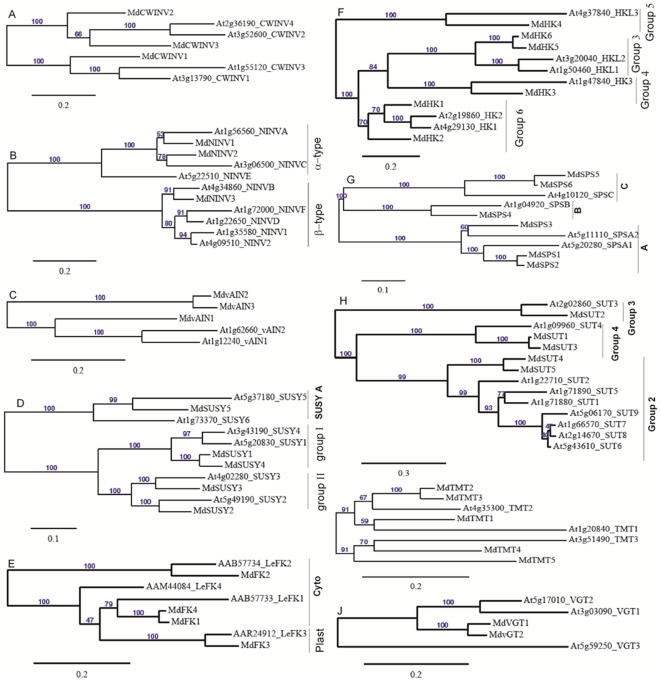
Maximum likelihood phylogeny of *Malus* genes encoding enzymes and transporters involved in sugar metabolism and accumulation with those from *Arabidopsis or Lycopersicon esculentum*. The tree was produced using MUSCLE and PhyML with the JTT amino acid substitution model, a discrete gamma model with 4 categories and an estimated shape parameter of 1.0. Bootstrapping was performed with 100 replicates. A, cell wall invertase (CWINV); B, neutral invertases (NINV), α and β type NINV according to Nonis et al. [37]; C, vacuolar acid invertase (vAINV); D; sucrose synthase (SUSY), different types according to Bieniawska et al. [38]; E, fructokinase (FK), cytosolic and plastid fructokinases in tomato according to Granot [19]; F, hexokinase (HK), different groups according to Karve et al. [39]; G, Sucrose phosphate synthases (SPS), *Arabidopsis* types according to Lutfiyya et al. [40]; H, Sucrose transporter (SUT), different groups according to Braun & Slewinski [41]; I, tonoplast monosaccharide transporter (TMT); J, vacuolar glucose transporter (vGT).

Blastp searches of the *Malus* Genome Database, using *A. thaliana invertase*, *SUSY*, *HK*, *SPS*, *SUT*, *TMT* and *vGT* sequences as query (except for *FK* using *L. esculentum*), allowed the identification of candidate genes in *Malus*, and these genes are expressed in *Malus* transcriptome based on their ESTs in Genbank. Nine genes encoding invertase were identified, including 3 *CWINVs*, 3 *NINVs* and 3 *vAINVs*. *MdCWIN1* had high similarity with *AtCWINV1* (At3g13790) and shared the same clade with *AtCWINV1* and *AtCWINV3* (At1g55120). Both *MdCWINV2* and *MdCWINV3* had high similarity and were in the same clade with *AtCWIN2* (At3g52600) and *AtCWINV4* (At2g36190) (Figure 2A). Both *MdNINV1* and *MdNINV2* shared high similarity of amino acid sequence, belonging to *Arabidopsis* ‘α-group’ with predicted mitochondrial or plastidic localization according to Nonis et al. [37], while *MdNINV3* was in ‘β-group’ with predicted cytosolic localization (Figure 2B). *MdvAINV1* was in the same clade with *AtvAINV1* (At1g12240) and *AtvAINV2* (At1g62660), whereas both *MdvAINV2* and *MdvAINV3* had high similarity and were in another clade (Figure 2C).

Five *SUSYs* were identified in *Malus* and their predicted localization had possibilities in cytosol, mitochondria or plastids (data not shown). Both *MdSUSY1* and *MdSUSY4* shared high homology and were in the same group with *AtSUSY1* (A5g20830) and *AtSUSY4* (At3g43190) according to Bieniawska et al. [38]. *MdSUSY2* and *MdSUSY3* were in the same phylogeny group with *AtSUSY2* (At5g49190) and *AtSUSY3* (At4g02280), respectively. However, *MdSUSY5* belonged to another *Arabidopsis SUSY* group: *SUSY* A [38] (Figure 2D).

Four orthologs of *FK* were found in *Malus* genome. *MdFK1* had high similarity with cytosol-localized *LeFK1* (AAB57733) and shared high similarity with *MdFK4*; *MdFK2* belonged to another clade with cytosol-localized *LeFK2* (AAB57734); *MdFK3* encoded a predicted plastid-localized *FK* (data not shown) and had the highest similarity with *LeFK3* (AAR24912) (Figure 2E).

Of the five ortholog of *HK* identified in *Malus*, both *MdHK1* and *MdHK2* had high homology with *AtHK1* (At4g29130) and *AtHK2* (At2g19860), respectively, and both belonged to *HK* ‘group 6’ according to Karve et al. [39]; *MdHK3* was an ortholog gene of *AtHK3* (At1g47840) and in the ‘group 4’ of *Arabidopsis HK* [39]; *MdHK4* was in the same clade with *AtHKL3* (At4g37840), which belonged to the ‘group 5’; *MdHK5* and *MdHK6* had high homology and were in the same clade with *AtHKL1* (At1g50460) and *AtHKL2* (At3g20040), both of which were in the ‘group 3’ [39] (Figure 2F).

Six *MdSPSs* identified in *Malus* covered 3 *Arabidopsis SPS* groups according to Lutfiyya et al. [40]. *MdSPS1* to *MdSPS3* belonged to the same group as *Arabidopsis AtSPSA1* (At5g20280) and *AtSPSA2* (At5g11110), and both *MdSPS1* and *MdSPS2* shared high similarity and had high homology with *AtSPSA1*, while *MdSPS3* was homologous to *AtSPSA2*. *MdSPS4* was an ortholog gene of *AtSPSB* (At1g04920) in another clade. Both *MdSPS5* and *MdSPS6* shared high similarity and were in the same group with *AtSPSC* (At4g10120) (Figure 2G).

### Candidate genes encoding sugar transporters

Five ortholog genes of *SUT* were identified in the *Malus* genome. *MdSUT1*, the same as *MdSUT1* (AY445915) reported by Fan et al. [16], shared high similarity with *MdSUT3* and had high homology with *AtSUT4*, which belonged to *SUC* ‘group 4’ according to Braun & Slewinski [41]; *MdSUT2* was in an independent clade with *AtSUT3* (At2g02860) in the ‘group 3’; Both *MdSUT4* and *MdSUT5* showed high similarity and had high homology with *AtSUT2* (At1g22710), which belonged to the ‘group 2’ [41] (Figure 2H).

Of the 5 ortholog genes of *TMT* identified in *Malus*, *MdTMT1*, *MdTMT2* and *MdTMT3* shared high similarity of amino acid sequence, and *MdTMT1* had high homology with *AtTMT1* (At1g20840) whereas both *MdTMT2* and *MdTMT3* had high homology with *AtTMT2* (At4g35300). *MdTMT4* showed high homology with *AtTMT3* (At3g51490) and were in the same clade with *MdTMT5* (Figure 2I).

Both *MdvGT1* and *MdvGT2* had high homology with *AtvGT1* (At3g03090) and *AtvGT2* (At5g17010), respectively, and were in the same clade, whereas they had low similarity with *AtvGT3* (At5g59250) (Figure 2J).

### Expression of genes in source and sink tissues

To determine tissue-specific expression levels of the candidate genes, qRT-PCR was used to analyze their mRNA relative expression abundance among mature leaves, shoot tips, young fruit (40 DAB) and mature fruit (135 DAB) (Table 1). *MdSDH1* and *MdSDH2*-9 were expressed mainly in fruit, with the expression level of *MdSDH2*-9 being 80 times higher in young fruit than in mature leaves. *MdSDH1* expression was higher in young fruit than in mature fruit, whereas the opposite was true for *MdSDH2*-9. *MdCWINV1* expression was much lower in fruit than in mature leaves and shoot tips. Both *MdCWINV2* and *MdCWINV3* transcript levels were much lower in mature fruit than in shoot tips, with similar expression level of *MdCWINV3* detected in shoot tips and young fruit. The expression levels of *MdNINV1* and *MdNINV2* were comparable among 4 different tissues, whereas *MdNINV3* had much higher expression in fruit than in other tissue types. All 3 *MdvAINVs* (especially *MdvAINV3*) had lower transcript levels in mature fruit, with the expression level of *MdvAINV1* being the highest in young fruit and the lowest in shoot tips. The expression levels of both *MdSUSY1* and *MdSUSY4* were lower in both young and mature fruit than in mature leaves, whereas those of both *MdSUSY2* and *MdSUSY3* were higher in young fruit than in mature leaves. *MdSUSY5* expression was higher in young fruit than in mature leaves, with the highest expression detected in shoot tips (5 times more than in mature leaves). The expression levels of *MdFKs* were either similar (*MdFK1, MdFK3* and *MdFK4*) or significantly higher (*MdFK2*) in shoot tips than in mature leaves; young fruit had comparable transcript abundance of *MdFK2* and *MdFK4*, but lower expression levels of *MdFK1 and MdFK3* than mature leaves; mature fruit had lower expression levels of *MdFK2-4*, but higher transcript abundance of *MdFK1*. The expression levels of *MdHK1* were comparable among 4 tissue types, but *MdHK2* and *MdHK3* had much higher transcript abundance in young fruit than in mature leaves, especially *MdHK3* (27.5 times higher). *MdHK4* expression was highest in shoot tips, but lowest in mature fruit. In contrast, both *MdHK5* and *MdHK6* had the highest expression in mature fruit. The transcript levels of all 6 *MdSPSs* were much higher in mature fruit than in both mature leaves and shoot tips. The expression levels of *MdSPS2*-6 were higher in mature fruit than in young fruit, but the opposite was true for *MdSPS1*. In addition, both *MdSPS5* and *MdSPS6* showed the lowest expression levels in shoot tips (Table 1).

**Table 1:** Comparison of relative mRNA expression for genes encoding enzymes involved in sugar metabolism (including *MdSDHs*, *MdCWINVs*, *MdNINVs*, *MdvAINVs*, *MdSUSYs*, *MdFKs*, *MdHKs*, and *MdSPSs*) among mature leaves, shoot tips, young fruit (40 DAB) and mature fruit (135 DAB) of apple.

	*MdSDH1*	*MdSDH2-9*	*MdCWINV1*	*MdCWINV2*	*MdCWINV3*	*MdNINV1*	*MdNINV2*
Mature leaves	1.00±0.33	1.00±0.25	1.00±0.18	1.00±0.26	1.00±0.23	1.00±0.19	1.00±0.18
Shoot tips	0.50±0.08	6.01±0.75	0.85±0.22	3.31±0.37	3.34±0.21	0.40±0.07	1.64±0.34
Young fruit	2.10±0.44	81.7±7.08	n/d	0.69±0.08	3.22±0.09	1.04±0.06	1.53±0.11
Mature fruit	17.6±2.01	49.2±6.83	0.03±0.01	0.06±0.01	0.02±0.01	0.60±0.13	0.73±0.12

	*MdNINV 3*	*MdvAINV1*	*MdvAINV 2*	*MdvAINV3*	*MdSUSY1*	*MdSUSY2*	*MdSUSY3*
Mature leaves	1.00±0.12	1.00±0.24	1.00±0.15	1.00±0.17	1.00±0.06	1.00±0.08	1.00±0.12
Shoot tips	1.57±0.27	0.17±0.01	1.09±0.10	1.84±0.13	0.78±0.07	1.73±0.09	1.54±0.23
Young fruit	8.76±0.89	7.45±1.21	1.13±0.05	0.21±0.14	0.34±0.07	2.10±0.23	2.15±0.16
Mature fruit	3.34±0.73	0.20±0.13	0.02±0.01	n/d	0.37±0.05	0.49±0.06	0.25±0.07

	*MdSUSY4*	*MdSUSY5*	*MdFK1*	*MdFK2*	*MdFK3*	*MdFK4*	*MdHK1*
Mature leaves	1.00±0.09	1.00±0.03	1.00±0.07	1.00±0.23	1.00±0.13	1.00±0.28	1.00±0.07
Shoot tips	0.31±0.03	5.98±0.56	1.12±0.09	3.81±0.42	0.97±0.15	1.25±0.34	0.75±0.17
Young fruit	0.17±0.02	2.22±0.32	0.31±0.11	1.39±0.12	0.49±0.04	1.58±0.15	1.66±0.20
Mature fruit	0.01±0.01	0.09±0.01	0.90±0.12	0.10±0.01	0.29±0.04	0.42±0.12	0.72±0.01

	*MdHK2*	*MdHK3*	*MdHK4*	*MdHK5*	*MdHK6*	*MdSPS1*	*MdSPS2*
Mature leaves	1.00±0.08	1.00±0.17	1.00±0.05	1.00±0.05	1.00±0.08	1.00±0.03	1.00±0.08
Shoot tips	1.29±0.09	4.17±0.33	2.63±0.13	0.57±0.07	0.71±0.09	3.07±0.12	1.07±0.09
Young fruit	4.71±0.43	27.5±4.53	0.95±0.18	0.83±0.08	2.42±0.31	47.8±4.39	3.11±0.85
Mature fruit	2.34±1.97	2.17±0.42	0.02±0.01	3.35±0.71	4.34±0.35	17.4±4.44	12.6±1.37

	*MdSPS3*	*MdSPS4*	*MdSPS5*	*MdSPS6*
Mature leaves	1.00±0.12	1.00±0.05	1.00±0.13	1.00±0.09
Shoot tips	0.78±0.11	1.41±0.13	0.02±0.00	0.06±0.01
Young fruit	3.18±0.61	1.01±0.27	0.06±0.02	1.14±0.10
Mature fruit	4.50±0.39	13.2±1.52	5.86±0.38	21.1±2.57

Values are means of three technical replicates of the reverse transcribed RNA sample pooled from 5 biological replicates ± SD. n/d means no expression was detected.

qRT-PCR was performed with gene-specific primers, except that a pair of universal primers was designed from the conserved cDNA region of *MdSDH2* to *MdSDH9* for the expression of *MdSDH2*-9. For each sample, transcript levels were normalized with those of *Actin*, and the relative expression levels of each gene were obtained using the ddCT method while expression in mature leaves was designated as ‘1’.

Both *MdSOT1* and *MdSOT2* showed higher transcript levels in sink organs than in mature leaves, except that mature fruit had similar level of *MdSOT2* as mature leaves (Table 2). Fruit had higher transcript levels of *MdSUT1-4*, but lower levels of *MdSUT5* than mature leaves and shoot tips. The expression levels of *MdTMT1* and *MdTMT2* were higher in fruit than in shoot tips and mature leaves, with the highest expression detected in mature fruit; the transcript levels of *MdTMT3*, *MdTMT4* and *MdTMT5* were highest in young fruit, but shoot tips also had higher expression levels of *MdTMT4* and *MdTMT5* than mature leaves. The expression levels of both *MdvGT1* and *MdvGT2* were higher in fruit than in shoot tips and mature leaves, with the highest expression detected in young fruit (Table 2).

**Table 2:** Comparison of relative mRNA expression for genes encoding sorbitol transporter (SOT), sucrose transporter (SUT), tonoplast monosaccharide transporter (TMT), and vacuolar glucose transporter (vGT) among mature leaves, shoot tips, young fruit (40 DAB) and mature fruit (135 DAB) of apple.

	*MdSOT1*	*MdSOT2*	*MdSUT1*	*MdSUT2*	*MdSUT3*	*MdSUT4*	*MdSUT5*
Mature leaves	1.00±0.24	1.00±0.13	1.00±0.15	1.00±0.08	1.00±0.11	1.00±0.18	1.00±0.09
Shoot tips	1.64±0.18	2.54±0.33	0.78±0.22	0.61±0.15	1.28±0.13	1.54±0.20	1.47±0.10
Young fruit	2.44±0.36	3.28±0.31	10.3±1.27	12.2±1.06	1.98±0.12	2.54±0.15	0.19±0.02
Mature fruit	3.07±0.53	1.02±0.04	4.50±1.46	2.95±0.13	2.58±0.43	1.90±0.32	0.48±0.04

	*MdTMT1*	*MdTMT2*	*MdTMT3*	*MdTMT4*	*MdTMT5*	*MdvGT1*	*MdvGT2*
Mature leaves	1.00±0.05	1.00±0.11	1.00±0.08	1.00±0.10	1.00±0.33	1.00±0.11	1.00±0.14
Shoot tips	0.62±0.09	1.41±0.07	2.33±0.13	10.0±1.30	33.3±3.00	1.31±0.23	1.57±0.38
Young fruit	2.10±0.44	1.83±0.22	30.5±3.85	33.6±3.57	212±16.3	6.27±0.69	8.49±1.16
Mature fruit	5.21±0.68	5.23±0.78	2.45±0.53	18.4±1.69	2.34±1.97	3.09±0.30	6.96±0.30

Values are means of three technical replicates of the reverse transcribed RNA sample pooled from 5 biological replicates ± SD.

qRT-PCR was performed with gene-specific primers. For each sample, transcript levels were normalized with those of *Actin*, and the relative expression levels of each gene were obtained using the ddCT method while expression in mature leaves was designated as ‘1.’

### Expression of genes during fruit development

**Figure 3:**
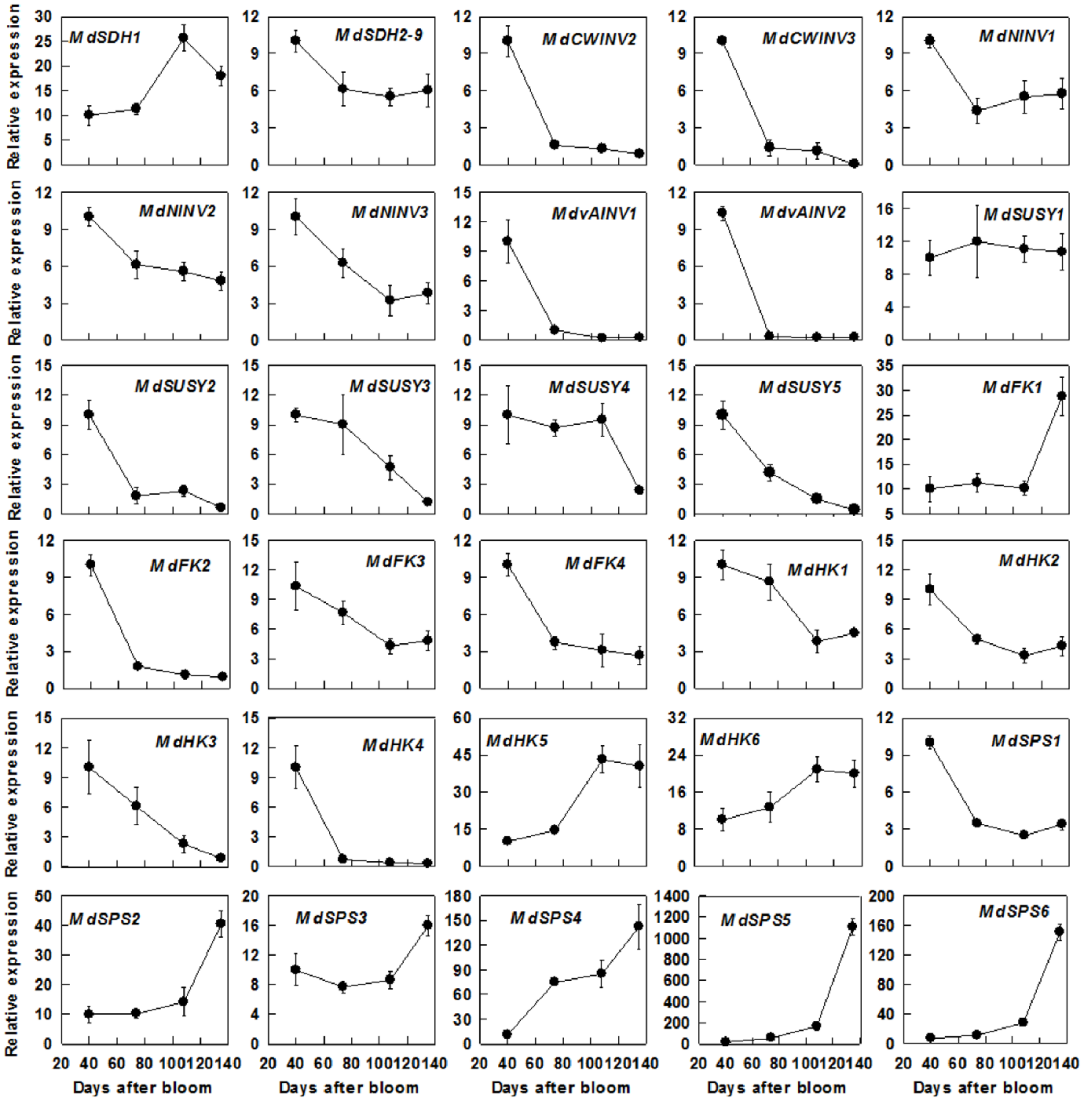
Relative mRNA expression for genes encoding enzymes involved in sugar metabolism (including *MdSDHs*, *MdCWINVs*, *MdNINVs*, *MdvAINVs*, *MdSUSYs*, *MdFKs*, *MdHKs*, *MdSPSs*) during apple fruit development. Quantitative RT-PCR was performed with gene-specific primers, except for *MdSDH2*-9 where a pair of universal primer was designed from the conserved cDNA region of *MdSDH2* to *MdSDH9*. For each sample, transcript levels were normalized with those of *Actin*, and the relative expression levels of each gene were obtained using the ddCT method while expression in 40-DAB-fruit was designated as ‘10’. Values are means of three replicates of the reverse transcribed RNA sample pooled from 5 biological replicates ± SD.

Transcript abundance of *MdSDH1* increased with fruit development from 40 DAB to 108 DAB and then decreased slightly towards maturity, whereas the expression level of *MdSDH2-9* showed a clear drop from 40 DAB to 74 DAB, and then remained unchanged to maturity (Figure 3). The expression levels of *MdCWINV1* and *MdvAINV3* were detected only in ripening fruit and 40-DAB-fruit, respectively (Data not shown). All of the transcript levels of *MdCWINV2*, *MdCWINV3*, *MdNINV1*, *MdNINV2*, *MdvAINV1* and *MdvAINV2* showed a dramatic decline from 40 DAB to 74 DAB, and then remained at low levels to maturity, whereas the transcript level of *MdNINV3* showed a continuous decrease from 40 DAB to 108 DAB (Figure 3). During fruit development, the expression level decreased from 40 DAB to 74 DAB for *MdSUSY2*, from 74DAB to maturity for *MdSUSY3*, from 108 DAB to maturity for *MdSUSY4*, and throughout the fruit development for *MdSUSY5*, whereas no change in expression level was observed for *MdSUSY1*. Relative mRNA expression levels of *MdFK1* remained unchanged until an obvious rise from 108 DAB to maturity. By contrast, *MdFK2* expression had a large drop from 40 DAB to 74 DAB, and then maintained at a low level to maturity. However, both *MdFK3* and *MdFK4* expression showed decreasing trends throughout fruit development (Figure 3). The expression levels of *MdHK1-4* decreased throughout fruit development, whereas the expression levels of both *MdHK5* and *MdHK6* increased towards fruit maturity. Except for a decrease in the transcript level observed for *MdSPS1* during fruit development, all the other 5 *MdSPSs* showed increases in their expression levels with fruit development, particularly *MdSPS5* and *MdSPS6* (Figure 3).

**Figure 4:**
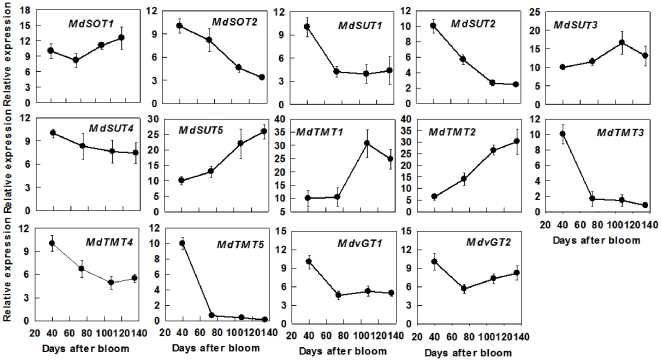
Relative mRNA expression for genes encoding sugar transporters (including *MdSOTs*, *MdSUTs*, *MdTMTs* and *MdvGTs*) during apple fruit development. Quantitative RT-PCR was performed with gene-specific primers. For each sample, transcript levels were normalized with those of *Actin*, and the relative expression levels of each gene were obtained using the ddCT method while expression in 40-DAB-fruit was designated as ‘10’. Values are means of three technical replicates of the reverse transcribed RNA sample pooled from 5 biological replicates ± SD.

The transcript level of *MdSOT1* showed a slight increase from 74 DAB to maturity, whereas that of *MdSOT2* decreased throughout fruit development (Figure 4). The transcript level of *MdSUT1* decreased from 40 DAB to 74 DAB and that of *MdSUT2* decreased throughout fruit development; those of both *MdSUT3* and *MdSUT4* remained relatively stable; whereas that of *MdSUT5* increased with fruit development. The transcript abundances of both *MdTMT1* and *MdTMT2* increased with fruit development, whereas those of *MdTMT3*, *MdTMT4* and MdTMT5 decreased during fruit development. The expression levels of both *MdvGT1* and *MdvGT2* dropped from 40 DAB to 74 DAB and remained unchanged to maturity (Figure 4).

### Activities of key enzymes in sugar metabolism during fruit development

**Figure 5:**
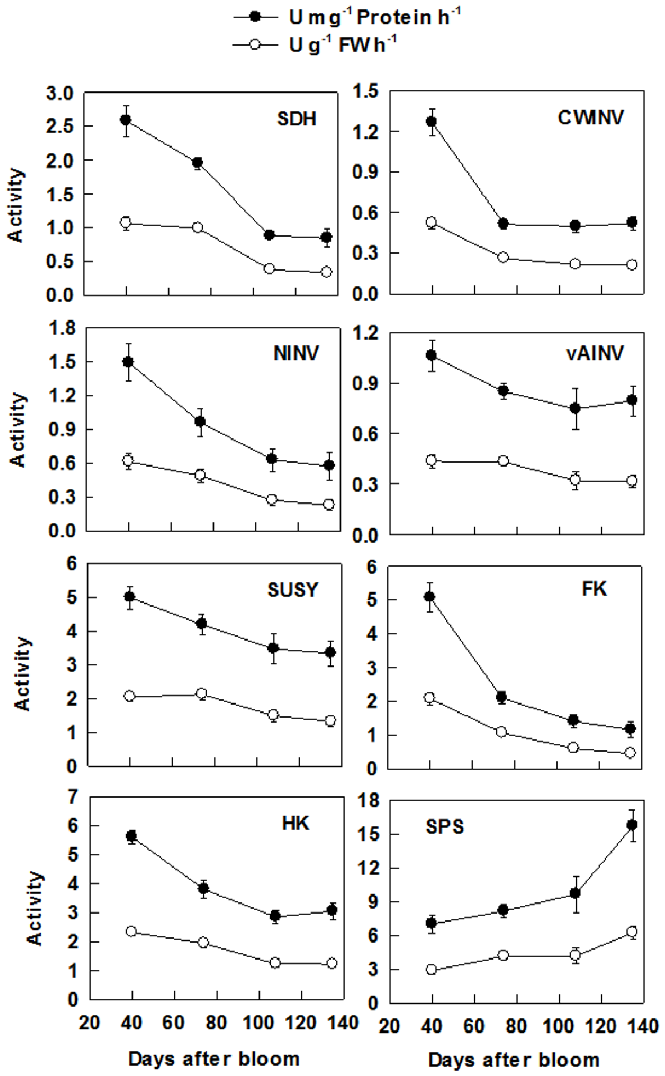
Activities of key enzymes involved in sugar metabolism during apple fruit development. SDH: Sorbitol dehydrogenase; CWINV: Cell wall invertase; NINV: Neutral invertase; vAINV: Vacuolar acid invertase; SUSY: Sucrose synthase; FK: Fructokinase; HK: Hexokinase; SPS: Sucrose-phosphate synthase. Values are means of five replicates ± SD.

On a soluble protein basis, the activity of SDH decreased from 40 to 108 DAB, with the activity at 108 and 135 DAB being about 40% of that at 40 DAB (Figure 5), but there was no correlation between the activity of SDH and the expression level of *SDH2-9* during fruit development (Table S8). The activity of CWINV decreased by 60% from 40 to 74 DAB and then remained unchanged to fruit maturity, which was correlated with the expression level of both *CWINV2* and *CWINV3* (Table S8). The activity of NINV decreased during fruit development (Figure 5), and was correlated with the expression level of both *NINV2* and *NINV3*. The activity of vAINV decreased from 40 to 108 DAB and then remained unchanged to fruit maturity, which was correlated with the expression level of both *vAINV1* and *vAINV2*. SUSY activity decreased with fruit development, and was correlated with the expression level of *SUSY5*. The activity of FK decreased with fruit development and was correlated with the expression level of *FK2*, *FK3* and *FK4*. The activity of HK also decreased with fruit development, which was correlated with the expression level of *HK2*, *HK3*, and *HK4*. In contrast, the activity of SPS increased slightly from 40 to 108 DAB, and then increased by about 63% to fruit maturity, which was correlated with the expression level of *SPS2*, *SPS5*, and *SPS6* (Table S8). When expressed on a fresh weight basis, the activities of all the enzymes showed similar trends as those on protein basis, but the degree of change was smaller (Figure 5).

### Concentrations of soluble sugars and starch during fruit development

**Figure 6:**
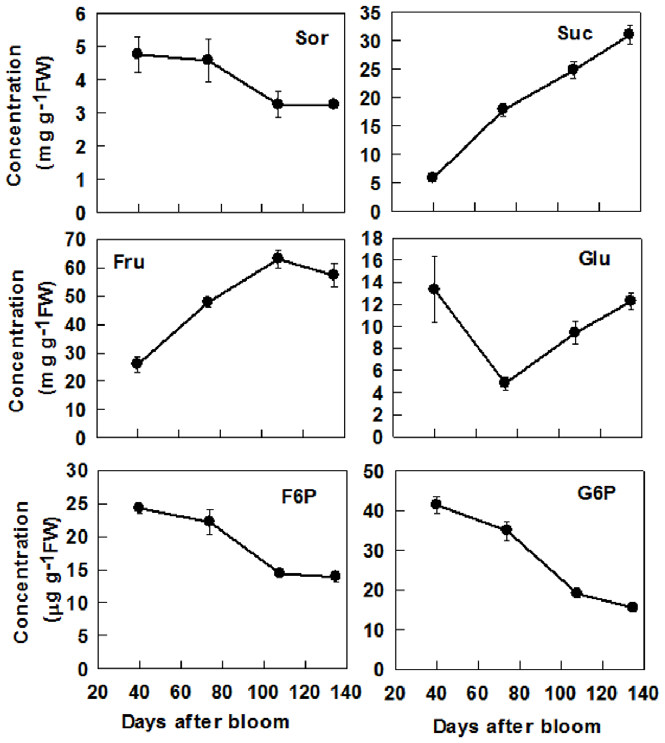
Concentrations of sorbitol (Sor), sucrose (Suc), fructose (Fru), glucose (Glc), fructose 6-phosphate (F6P) and glucose 6-phosphate (G6P) during apple fruit development. Values are means of five replicates ± SD.

**Figure 7:**
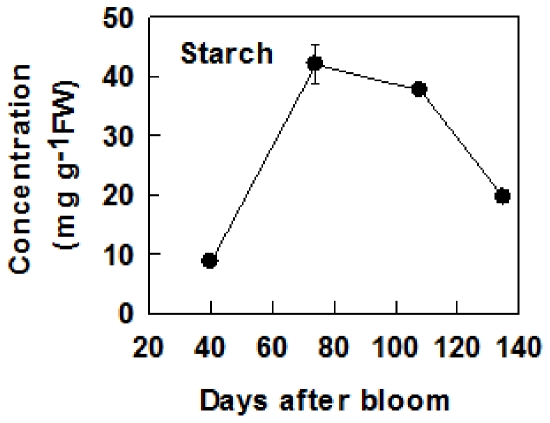
Starch concentrations during apple fruit development. Values are means of five replicates ± SD.

Sor concentration decreased with fruit development (Figure 6). Suc concentration increased nearly 5 times from 40 DAB to maturity (Figure 6). Fru concentration increased from 40 to 108 DAB, and dropped slightly at maturity, whereas Glc concentration decreased from 40 to 74 DAB, and then increased toward maturity. Both F6P and G6P decreased with fruit development (Figure 6). Of all the soluble sugars, Fru concentration was the highest in mature fruit, which was about 2 times that of Suc and 4 times that of Glc. Starch concentration increased rapidly from 40 to 74 DAB, was constant at the highest level from 74 to 108 DAB, and then decreased towards maturity (Figure 7).

## Discussion

### Sugar metabolism and accumulation is developmentally regulated in apple

Our data clearly indicate that the metabolism of sugars in fruit is highly regulated by developmental processes. At the early stage of fruit development (40 DAB), high activities of SDH, NINV, SUSY, FK and HK found in this study and reported previously by Chourey and Berüter [17] match the high expression levels of their genes (Figures 3, 5). This makes rapid metabolism of the imported sugars possible to satisfy the requirement for energy and intermediates by cell division and growth at the early stage of fruit development. As a result, the concentrations of Fru, Suc and starch are low (Figure 6). As the requirement for energy and carbon skeletons decreases with fruit development, the expression levels and the activities of these enzymes decrease (Figures 3, 5), resulting in a smaller proportion of the imported sugars metabolized in the glycolysis/TCA cycle and subsequent amino acid and protein synthesis. Consequently, Fru and Suc accumulate in the vacuole [13] and starch synthesis is up-regulated to allow the storage of the extra imported sugars in the insoluble form in plastids [1]. At the late stage of fruit development, starch breaks down (Figure 6), and sucrose continues to accumulate, which elevates the total concentrations of soluble sugars, and the sweetness of the fruit to a maximum at maturity. From an evolutionary perspective, the maximum accumulation of soluble sugars at fruit maturity helps attract animals and human for seed dispersal.

Most Fru and Glc are stored in the central vacuole of parenchyma cells in apple, which occupies more than 80% of the cell's volume [13], [42]. Rapid accumulation of Fru during apple cell expansion results from the coordinated actions of three factors. First, abundant Fru is generated from sorbitol and sucrose. Although the transcripts and activities of SDH, invertase and SUSY decreased with fruit development (Figure 5), most sorbitol has been converted to Fru as indicated by its low concentration in fruit (Figure 6). Second, decreased expression and activity of FK (Figures 3, 5) indicates less Fru is metabolized with fruit development and more is available for accumulation. Finally, up-regulation of carrier proteins that are localized on the tonoplast membrane suggests active transport of Fru from cytosol into the vacuole is enhanced during fruit development. Although it remains unknown whether Fru transporters are present on the tonoplast membrane, vacuolar carrier proteins encoded by *TMT*s transport both Glc and Fru [43]. As reported in grape [11], 5 orthologs of *TMT* are present in the apple genome and all had much higher expression in fruit than in mature leaves and shoot tips (Table S6; Figure 4). The transcript levels of *MdTMT1* and *MdTMT2*, both of which shared high amino acid similarity with *AtTMT1* (Figure 2), showed similar developmental trends as Fru concentration (Figures 4, 6), suggesting both proteins may be involved in transporting Fru into the vacuole.

Glucose is primarily derived from the hydrolysis of the imported Suc via CWINV and NINV before significant starch breakdown occurs in fruit. Considering that phloem unloading of sorbitol and Suc involves an apoplastic step between SE-CC complexes and parenchyma cells in apple fruit [14], and sorbitol transporters are competitively inhibited by Glc and Fru [44], the hydrolysis of Suc by cell wall invertase is expected to operate only on a small scale to avoid excessive accumulation of sorbitol in the apoplast. Viewed this way, the majority of the unloaded Suc from SE-CC may be directly taken up into parenchyma cells by Suc transporters on the plasma membrane, and subsequently hydrolyzed via NINV. Decreases in the expression and activities of both CWINV and NINV (Figures 3, 5) indicate that the generation of Glc decreases with fruit development. At the same time, however, the decreased HK activity suggests that less Glc is metabolized as well. When starch breaks down rapidly towards maturity (Figure 7), Glc concentration increased only slightly (Figure 6). The developmental changes of Glc concentrations are consistent with the expression patterns of *MdvGTs* (Figure 4), and it has been confirmed that *AtvGT1* is a specific Glc transporter located on the tonoplast membrane and has an important function in seed germination and flowering of *Arabidopsis* [45]. These results suggest that *MdvGTs* may play a role in transporting Glc into vacuole in apple fruit. In addition, the expression level of *MdTMT3*, which has high similarity with *MdTMT2* (Figure 2), is consistent with high Glc concentration during early fruit development. Although *AtTMT3* is hardly expressed at any stage of the plant's life cycle [43], the expression levels of *MdTMT4* and *MdTMT5*, both of which belonged to the same clade as *AtTMT3* (Figure 2), were much higher in sink tissues than in mature leaves, especially *MdTMT5* in 40-DAB-fruit (Figure 4). Further work is needed to clarify their roles in sugar accumulation in apple fruit.

Besides hexoses, Suc also accumulates to a high concentration in the ripening apple fruit, although in young fruit a large proportion of Suc is in the apoplast and cytosol [42]. There are two sources of sucrose for accumulation: the imported sucrose that has not been metabolized and newly synthesized sucrose. The decreased transcript levels and activities of invertase and SUSY with fruit development (Figures 3, 5) are consistent with the accumulation pattern of Suc before rapid starch breakdown. The continued accumulation of Suc towards maturity is tightly associated with the rapid increase in the transcript levels and activities of SPS (Figures 3, 5). This suggests that newly synthesized Suc via SPS contributes significantly to the total Suc level at maturity. So, Suc accumulation in apple fruit approaching maturity is of SPS type according to the categorization of mechanisms controlling sugar accumulation in fruit by Yamaki [46], as in melon and strawberry [8], [47]. The glucose released from starch breakdown appears to contribute to Suc synthesis because Glc showed only a slight increase towards maturity (Figure 6). Although 5 orthologs of *SUTs* were identified in *Malus* genome (Figure 2), all are Suc/H^+^ symporters, which transport Suc into cytosol from apoplast or vacuole. So far, not a single Suc/H^+^ antiporter that transports Suc into vacuole has been identified in plants [41], [48]. However, it was reported recently that both TMT1 and TMT2 also transport Suc across the tonoplast membrane in *Arabidopsis* [49]. The expression patterns of both *MdTMT1* and *MdTMT2* (Figure 4) are in general agreement with that of Suc accumulation in fruit (Figure 6). Once Suc is inside the vacuole, it can be converted to Glc and Fru by vAINV. By changing the relative contribution of sugars, vAINV regulates the elongation of cotton fiber and *Arabidopsis* root in an osmotic dependent and independent manner, respectively [50]. However, the expression levels of 3 *MdvAINVs* were very low during apple fruit development except at 40 DAB, which is consistent with sustained Suc accumulation from cell expansion to fruit maturity (Figures 3, 5).

### Differential expression patterns of multiple members of gene families for key enzymes involved in sugar metabolism suggest different roles in sugar metabolism

Each enzyme involved in Suc-Suc cycle is encoded by a family of genes that are differentially expressed in mature leaves, shoot tips and fruit. Because sugar metabolism and accumulation is tissue type-dependent, comparison of gene expression patterns, enzyme activities, and sugar metabolism and accumulation allows us to gain insights into the possible functions of these genes.

CWINV is typically considered as a sink-specific enzyme, and its activity is usually low in source leaves [51], [52]. However, all 3 *MdCWINVs* identified in apple had lower expression levels in fruit than in leaves, except for *MdCWINV3* in 40 DAB-fruit (Table 1, Figure 3). Since the unloading of sucrose in shoot tips is symplastic [53], the high expression level of *MdCWINV3* in both shoot tips and in young fruit suggests that this isoform of *CWINV* may play a role in cell division or cell growth [5], [54], and may not necessarily be related to apoplastic unloading of Suc in fruit [1]. The much higher expression level of *MdCWINV2* in shoot tips than in fruit (Table 1) suggests that the role of this isoform in Suc unloading might be very small, if any.

The expression levels of *MdNINV1* and *MdNINV2*, both of which belong to the α-group with a predicted mitochondrial and plastidic localization respectively (Figure 2), were comparable in different tissue types (Table 1). By contrast, *MdNINV3*, a predicted cytosolic NINV that has high homology with *Arabidopsis* cytosolic *AtNINV* genes: *AtNINV1*/*AtNINV2* [55], had much higher transcript abundance in fruit than in leaves and shoot tips (Table 1, Figure 2), and its expression levels matched well with NINV activity during fruit development (Figure 3). These results suggest that *MdNINV3* may play an important role in controlling Suc concentration in the cytosol of apple fruit.

In sinks where Suc is the only imported carbon, such as potato tubers [56], maize kernels [57] and kiwifruit fruit [30], SUSY activity is correlated with the sink strength of storage organs. In apple where Suc accounts for only about one third of the imported carbon, the expression levels of *MdSUSY1* and *MdSUSY4* were lower in fruit and shoot tips than in mature leaves, whereas the expression levels of other *MdSUSY*s in fruit were ∼2 times of those in mature leaves (except for that *MdSUSY5* was much higher in shoot tips) (Table 1). Increased *MdSUSY1* expression has been observed in the shoot tips of transgenic apple with decreased Sor supply but increased Suc supply from leaves, and both *MdSUSY1* transcript level and SUSY activity were dramatically enhanced by Suc feeding [10]. *MdSUSY1* had a relatively stable expression during fruit development, which is consistent with the finding of Janssen et al. [7], whereas *MdSUSY4* expression showed a great drop towards fruit maturity. The expression pattern of neither *MdSUSY1* nor *MdSUSY4* is consistent with decreased SUSY activities during apple fruit development (Figures 3, 5). In contrast, the expression patterns of *MdSUSY2*, *MdSUSY3* and *MdSUSY5* showed general agreement with decreased SUSY activities during fruit development (Figures 3, 5). These results suggest that *MdSUSY1* may play an important role in shoot tips whereas *MdSUSY2*, *MdSUSY3* and *MdSUSY5* may be largely responsible for the total SUSY activities in apple fruit.

Since more than 80% of the carbon flux goes through Fru in apple sink cells compared with only 50% in other plants where Suc is the only form of imported carbon, apple sinks that do not accumulate Fru significantly (both shoot tips and young fruit) are expected to have a stronger FK activity for Fru utilization than those that actively accumulate Fru (e.g. fruit during cell expansion). Consistent with this idea, the expression patterns of *MdFKs* were similar (*MdFK1*, *MdFK3*, and *MdFK4*) or significantly higher (*MdFK2*) in shoot tips than in mature leaves, and were higher (*MdFK2-4*) in young fruit than in mature fruit (Table 1, Figure 3). The higher expression of *MdFK2* (ortholog of cytosol-localized *LeFK2* [19]) in shoot tips than in young fruit (Table 1, Figure 2), with dramatic decline in the expression level in fruit at 74 DAB (Figure 3) suggests that it may play an important role in efficient utilization of Fru in both shoot tips and young fruit, and Fru accumulation during fruit cell expansion. The up-regulation of the expression of *MdFK1* towards fruit maturity (Figure 3) appears to be consistent with phosphorylation of Fru for Suc synthesis.

Six HK genes were identified in apple in this study, which is similar to the number of *HK*s found in rice and *Arabidopsis* [12], [39]. HK has an important role in regulating carbon flow and energy status of the cell [57]. Both *MdHK1* and *MdHK2* are homologous to *AtHK1* and *AtHK2*, respectively (Figure 2), and were predicted to target the secretory pathway while *AtHK1* and *AtHK2* are located on the outer mitochondrial membrane [39]. Transcript abundance of *MdHK1* was comparable in different tissue types of apple, whereas *MdHK2* had a higher expression level in fruit than in other tissues (Table 1). However, both expression levels decreased with fruit development (Figures 3, 6, 7), suggesting that both *MdHK1* and *MdHK2* may be more related to the modulation of glycolytic flux and energy status of the cell in young fruit [57]. *MdHK3*, as a predicted plastid localized protein (data not shown), is an ortholog gene of *AtHK3* (a plastid enzyme, [39]), and much higher expression level of *MdHK3* was observed during rapid starch accumulation (Figure 3). It remains unclear whether the protein encoded by *MdHK3* plays a role in maintaining Glc concentration or controlling energy status in plastid, but it has been suggested that *AtHK3* might only have a catalytic function [39]. Of the three HK genes in apple that are orthologs of the *Arabidopsis AtHK1*-*3*, *MdHK4*, an ortholog of *AtHKL3*, which is thought not to bind Glc [39], had a very low expression level in fruit after 74 DAB (Figure 3). *MdHK5* and *MdHK6* are in the same phylogeny clade with *AtHKL1* and *AtHKL2* (Figure 2), both of which were predicted to bind Glc with a relatively lower affinity than *AtHK1* [39]. Karve and Moore [58] suggested that *AtHKL1* functions as a negative regulator that limits plant growth under excessive Glc availability. Higher expression levels of *MdHK5* and *MdHK6* towards fruit maturity seem to imply that they may be related to fast utilization of the Glc released from starch breakdown.

The 6 *MdSPSs* identified in apple (Figure 2) cover all three main groups of plant *SPSs* [40]. *MdSPS1-3* are in the same group as *Arabidoipsis AtSPSA1* and *AtSPSA2* (Figure 2). Both *MdSPS1* and *MdSPS2* share high homology of amino acid sequence, and are otholog genes of *AtSPSA1*, the main gene responsible for SPS activity in *Arabidopsis* leaves [59]. All *MdSPS1-3* had higher expression in fruit than in mature leaves (Table 1). However, decreases in the expression of *MdSPS1* with fruit development suggest that *MdSPS1* might not be involved in Suc accumulation in apple fruit towards maturity. In contrast, higher expression levels of *MdSPS2* and *MdSPS3* towards fruit maturity are consistent with elevated SPS activity and fast accumulation of Suc during this period (Figures 3, 6). It has also been reported that the transcript level of *CmSPS1*, an ortholog gene of *AtSPSA1* and *MdSPS2* in melon, shows the same trend as SPS enzyme activity and Suc accumulation in melon fruit [8]. Although a mutant of *AtSPSA2*, an ortholog gene of *MdSPS3*, has no significant effect on SPS enzyme activity in *Arabidopsis* [59], the expression of *MdSPS3* was also higher towards fruit maturity (Figure 3), which is similar to the result of a microarray analysis that EST sequence (EB123469) of *MdSPS3* also showed up-regulation in mature apple fruit [7]. The transcript level of *MdSPS4*, an ortholog gene of *AtSPSB* whose mutant has no significant effect on SPS enzyme activity in *Arabidopsis* [59], was correlated with Suc concentration in apple (Figures 3, 6). Both *MdSPS5* and *MdSPS6* share high similarity and are in the same group with *AtSPSC* (Figure 2). Although a mutation in *AtSPSC* only caused a 13% decrease in SPS activity in *Arabidopsis* [59], the expression levels of both *MdSPS5* and *MdSPS6* were dramatically up-regulated in fruit towards maturity (Figure 3) whereas their expression levels were very low in shoot tips that do not accumulate significant amount of Suc (Table 1). This suggests that both *MdSPS5* and *MdSPS6* may play important roles in elevating SPS activity for Suc accumulation towards fruit maturity.

Significant correlations between the expression level of a member of a gene family and the total activity of its enzyme were taken as evidence for transcriptional regulation of enzyme activity during fruit development and were also used as a tool for identifying genes that play a role in controlling sugar metabolism in this study. However, it should be pointed out that the in vivo activity of many enzymes is also regulated at the posttranslational level, for example, SPS is regulated by protein phosphorylation and allosteric effects of G6P and inorganic phosphate [60]. The lack of a significant correlation between the expression level of members of *MdSDHs* and total SDH activity suggests that postranslational regulation might play a role in determining SDH activity.

### Conclusions

Sugar metabolism in apple fruit is developmentally regulated to match the high requirements for energy and intermediates during the early stage of fruit development and sugar accumulation from the beginning of cell expansion to fruit maturity. At the early stage of fruit development, imported sorbitol and Suc are rapidly metabolized by high activities of SDH, invertase, SUSY, FK and HK to satisfy the requirement for energy and intermediates by cell division and growth. As fruit cell expansion continues, the requirement for energy and carbon skeleton decreases, and the activities of these enzymes are down-regulated. Decreased FK activity makes Fru available for accumulation in the vacuole while up-regulation of starch synthesis allows extra carbon stored in the insoluble form in plastids. At the late stage of fruit development, starch breaks down and up-regulation of sucrose synthesis via SPS contributes significantly to the continued Suc accumulation in the vacuole, elevating the total soluble sugars to a maximum at maturity. In addition, special transporters, e.g. proteins encoded by *MdTMT1* and/or *MdTMT2*, may play important roles in controlling the accumulation of Fru and Suc in apple fruit. From an evolutionary perspective, accumulation of high concentrations of soluble sugars at maturity determines the sweetness of fruit, which serve as attractants for seed dispersal by animals, while low soluble sugars and high starch make immature fruit unsavory to protect seeds from animals or other herbivores.

This work represents a comprehensive analysis of genes involved in sugar metabolism and accumulation in apple. Comparison of the expression patterns of multigene families in mature leaves, shoot tips and fruit, and with those homologous genes functionally characterized in *Arabidopsis* and other plants allow us to gain insights into their functions in controlling sugar homeostasis and accumulation during apple fruit development. The genes identified in this study, such as *MdFK1*, *MdFK2*, *MdNINV3*, *MdSPS5*, *MdSUT4*, *MdTMT1*, *MdTMT2* etc., and their expression data presented here will serve as a platform for further studies to understand sugar metabolism and accumulation in fruit and to manipulate sugar metabolism for improvement of fruit quality related to carbohydrates.

## Supporting Information

Table S1 **Information of invertase genes identified in apple including cell wall invertase (CWINV), neutral invertase (NINV), and vacuole acid invertase (vAINV).**
(DOC)

Table S2 **Information of sucrose synthase (SUSY) genes identified in apple.**
(DOC)

Table S3 **Information of fructokinase (FK) genes identified in apple.**
(DOC)

Table S4 **Information of hexokinase (HK) genes identified in apple.**
(DOC)

Table S5 **Information of sucrose-phosphate synthase (SPS) genes identified in apple.**
(DOC)

Table S6 **Information of sucrose transporter (SUT), tonoplast monosaccharide transporter (TMT) and vacuole glucose transporter (vGT) genes identified in apple.**
(DOC)

Table S7 **Oligonucleotide sequences for primers used in this study.**
(DOC)

Table S8 **Linear regression equations between enzyme activity (y, µmol mg^−1^ Protein h^−1^) and the relative expression level (x) of its genes, y = ax+b, during apple fruit development.**
(DOC)
