# Supplementary material for: Expression Patterns of Genes Involved in Sugar Metabolism and Accumulation during Apple Fruit Development
Source: PLoS One. 2012 Mar 7;7(3):e33055. doi: 10.1371/journal.pone.0033055 (PMC3296772; doi:10.1371/journal.pone.0033055)
Supplement: Table S1 — Information of invertase genes identified in apple including cell wall invertase (CWINV), neutral invertase (NINV), and vacuole acid invertase (vAINV). (DOC) [file pone.0033055.s001.doc]

**Table S1** Information of invertase genes identified in apple including cell wall invertase (CWINV), neutral invertase (NINV), and vacuole acid invertase (vAINV)

|  | Size  n.t.  (bp)/a.a | *Malus domestica* genome | | *Malus* EST sequence  (Similarity more than 98%) | | Homologous genes | | | |
| --- | --- | --- | --- | --- | --- | --- | --- | --- | --- |
|  |  | Position on Chr | Gene ID |  |  | In *Arabidopsis* | | In *Vitis vinifera* | |
|  |  |  |  |  |  | Locus in TAIR | % similarity (a.a.) | Gene ID in Genbank | % similarity (a.a.) |
| *MdCWINV1* | 1716  /571 | chr13:29305782..29311850 | MDP0000010638 | [EB152734](http://www.ncbi.nlm.nih.gov/nucleotide/91042316?report=genbank&log$=nucltop&blast_rank=1&RID=T9ZVPM2C011)  [DR989875](http://www.ncbi.nlm.nih.gov/nucleotide/71812484?report=genbank&log$=nucltop&blast_rank=2&RID=T9ZVPM2C011)  [GO560741](http://www.ncbi.nlm.nih.gov/nucleotide/226804441?report=genbank&log$=nucltop&blast_rank=3&RID=T9ZVPM2C011)  [DT002064](http://www.ncbi.nlm.nih.gov/nucleotide/71824672?report=genbank&log$=nucltop&blast_rank=4&RID=T9ZVPM2C011)  [GO527601](http://www.ncbi.nlm.nih.gov/nucleotide/226772395?report=genbank&log$=nucltop&blast_rank=5&RID=T9ZVPM2C011) | [EB152430](http://www.ncbi.nlm.nih.gov/nucleotide/91042012?report=genbank&log$=nucltop&blast_rank=6&RID=T9ZVPM2C011)  [CO867438](http://www.ncbi.nlm.nih.gov/nucleotide/51097588?report=genbank&log$=nucltop&blast_rank=8&RID=T9ZVPM2C011)  [EB151643](http://www.ncbi.nlm.nih.gov/nucleotide/91041225?report=genbank&log$=nucltop&blast_rank=9&RID=T9ZVPM2C011)  [EG025846](http://www.ncbi.nlm.nih.gov/nucleotide/115342594?report=genbank&log$=nucltop&blast_rank=11&RID=T9ZVPM2C011) | At3g13790  (*AtCWINV1*) | 66.4 | LOC100232951  (*VvCWINV*) | 72.0 |
| *MdCWINV2* | 1734/577 | chr12:2618551..2623108 | MDP0000275150 | GO543053  GO514202  DY255328 | CN996772  GO551577  GO547699 | At3g52600  (*AtCWINV2*) | 59.9 | LOC100263168 | 73.7 |
| *MdCWINV3* | 1833/610 | chr14:2743616..2747685 | MDP0000268052 | [GO511990](http://www.ncbi.nlm.nih.gov/nucleotide/226756209?report=genbank&log$=nucltop&blast_rank=1&RID=TA2WF4J0015)  [GO502889](http://www.ncbi.nlm.nih.gov/nucleotide/226745321?report=genbank&log$=nucltop&blast_rank=2&RID=TA2WF4J0015)  [GO502771](http://www.ncbi.nlm.nih.gov/nucleotide/226747401?report=genbank&log$=nucltop&blast_rank=3&RID=TA2WF4J0015)  [GO518228](http://www.ncbi.nlm.nih.gov/nucleotide/226763388?report=genbank&log$=nucltop&blast_rank=4&RID=TA2WF4J0015) | [GO514229](http://www.ncbi.nlm.nih.gov/nucleotide/226759160?report=genbank&log$=nucltop&blast_rank=6&RID=TA2WF4J0015)  [CN580084](http://www.ncbi.nlm.nih.gov/nucleotide/46991634?report=genbank&log$=nucltop&blast_rank=8&RID=TA2WF4J0015)  [CO066037](http://www.ncbi.nlm.nih.gov/nucleotide/48735518?report=genbank&log$=nucltop&blast_rank=9&RID=TA2WF4J0015) | At3g52600  (*AtCWINV2*) | 61.1 | LOC100263168 | 68.2 |
| *MdNINV1* | 1998/665 | chr4:18807745..18811112 | MDP0000652278 | [EB142114](http://www.ncbi.nlm.nih.gov/nucleotide/91031696?report=genbank&log$=nucltop&blast_rank=1&RID=KYWB89HW014)  [EB151786](http://www.ncbi.nlm.nih.gov/nucleotide/91041368?report=genbank&log$=nucltop&blast_rank=2&RID=KYWB89HW014) | [EB123882](http://www.ncbi.nlm.nih.gov/nucleotide/91013464?report=genbank&log$=nucltop&blast_rank=7&RID=KYWB89HW014)  [EB122192](http://www.ncbi.nlm.nih.gov/nucleotide/91011774?report=genbank&log$=nucltop&blast_rank=8&RID=KYWB89HW014) | At1g56560  (*AtNINVA*) | 71.8 | LOC100254513 | 77.8 |
| *MdNINV2* | 2094/697 | chr16:8043095..8046825 | MDP0000133399 | [EB111480](http://www.ncbi.nlm.nih.gov/nucleotide/91001059?report=genbank&log$=nucltop&blast_rank=1&RID=KYY7PXXJ016)  [EB123368](http://www.ncbi.nlm.nih.gov/nucleotide/91012950?report=genbank&log$=nucltop&blast_rank=3&RID=KYY7PXXJ016)  [CX024921](http://www.ncbi.nlm.nih.gov/nucleotide/56434609?report=genbank&log$=nucltop&blast_rank=5&RID=KYY7PXXJ016) | [DT042383](http://www.ncbi.nlm.nih.gov/nucleotide/71923237?report=genbank&log$=nucltop&blast_rank=7&RID=KYY7PXXJ016)  EB121908 | At3g06500  (*AtNINVC*) | 66.6 | LOC[100233037](http://www.ncbi.nlm.nih.gov/sites/entrez?db=gene&cmd=Retrieve&dopt=full_report&list_uids=100233037)  (*VvNINV*) | 77.7 |
| *MdNINV3* | 1719/572 | chr5:22192295..22195888 | MDP0000596702 | CV[628130](http://www.ncbi.nlm.nih.gov/nucleotide/54620994?report=genbank&log$=nucltop&blast_rank=1&RID=KYTJDGR0014)  [EB127152](http://www.ncbi.nlm.nih.gov/nucleotide/91016734?report=genbank&log$=nucltop&blast_rank=2&RID=KYTJDGR0014)  EB132132  [EB131869](http://www.ncbi.nlm.nih.gov/nucleotide/91021451?report=genbank&log$=nucltop&blast_rank=5&RID=KYTJDGR0014)  [GO498732](http://www.ncbi.nlm.nih.gov/nucleotide/226743187?report=genbank&log$=nucltop&blast_rank=6&RID=KYTJDGR0014) | [EB132944](http://www.ncbi.nlm.nih.gov/nucleotide/91022526?report=genbank&log$=nucltop&blast_rank=8&RID=KYTJDGR0014)  [EB134440](http://www.ncbi.nlm.nih.gov/nucleotide/91024022?report=genbank&log$=nucltop&blast_rank=10&RID=KYTJDGR0014)  [EB134028](http://www.ncbi.nlm.nih.gov/nucleotide/91023610?report=genbank&log$=nucltop&blast_rank=12&RID=KYTJDGR0014)  [EB133058](http://www.ncbi.nlm.nih.gov/nucleotide/91022640?report=genbank&log$=nucltop&blast_rank=13&RID=KYTJDGR0014)  [DT003334](http://www.ncbi.nlm.nih.gov/nucleotide/71825942?report=genbank&log$=nucltop&blast_rank=14&RID=KYTJDGR0014) | At4g34860  (*AtNINVB*) | 82.1 | LOC100253759 | 83.4 |
| *MdvAINV1* | 2037/678 | chr6:9161660..9166433 | MDP0000124776 | [CO904733](http://www.ncbi.nlm.nih.gov/nucleotide/51295036?report=genbank&log$=nucltop&blast_rank=1&RID=M9C0TZZJ01S)  [CV081389](http://www.ncbi.nlm.nih.gov/nucleotide/51559944?report=genbank&log$=nucltop&blast_rank=2&RID=M9C0TZZJ01S) | [CV083395](http://www.ncbi.nlm.nih.gov/nucleotide/51562472?report=genbank&log$=nucltop&blast_rank=6&RID=M9C0TZZJ01S)  [GO558995](http://www.ncbi.nlm.nih.gov/nucleotide/226803152?report=genbank&log$=nucltop&blast_rank=10&RID=M9C0TZZJ01S) | At1g12240  (*AtvAINV1*) | 64.6 | LOC100241232 | 67.4 |
| *MdvAINV2* | 1929/642 | chr7:22464359..22468029 | MDP0000793093 | [GO502615](http://www.ncbi.nlm.nih.gov/nucleotide/226745846?report=genbank&log$=nucltop&blast_rank=1&RID=TAB33EER015)  [CN579127](http://www.ncbi.nlm.nih.gov/nucleotide/46990677?report=genbank&log$=nucltop&blast_rank=3&RID=TAB33EER015)  [GO503416](http://www.ncbi.nlm.nih.gov/nucleotide/226748283?report=genbank&log$=nucltop&blast_rank=4&RID=TAB33EER015)  [CN996957](http://www.ncbi.nlm.nih.gov/nucleotide/48488847?report=genbank&log$=nucltop&blast_rank=26&RID=TAB33EER015) | [CN491123](http://www.ncbi.nlm.nih.gov/nucleotide/46607441?report=genbank&log$=nucltop&blast_rank=6&RID=TAB33EER015)  [GO512067](http://www.ncbi.nlm.nih.gov/nucleotide/226756673?report=genbank&log$=nucltop&blast_rank=12&RID=TAB33EER015)  [EB146276](http://www.ncbi.nlm.nih.gov/nucleotide/91035858?report=genbank&log$=nucltop&blast_rank=18&RID=TAB33EER015) | At1g12240  (*AtvAINV1*) | 56.3 | LOC100256970 | 62.6 |
| *MdvAINV3* | 1938/645 | chr1:23870970..23875107 | MDP0000258465 | [GO505468](http://www.ncbi.nlm.nih.gov/nucleotide/226749678?report=genbank&log$=nucltop&blast_rank=1&RID=TACDPN2N01S)  [GO544158](http://www.ncbi.nlm.nih.gov/nucleotide/226787148?report=genbank&log$=nucltop&blast_rank=2&RID=TACDPN2N01S)  [EB126722](http://www.ncbi.nlm.nih.gov/nucleotide/91016304?report=genbank&log$=nucltop&blast_rank=3&RID=TACDPN2N01S)  [EB122193](http://www.ncbi.nlm.nih.gov/nucleotide/91011775?report=genbank&log$=nucltop&blast_rank=4&RID=TACDPN2N01S)  [GO561080](http://www.ncbi.nlm.nih.gov/nucleotide/226805188?report=genbank&log$=nucltop&blast_rank=6&RID=TACDPN2N01S) | [GO512077](http://www.ncbi.nlm.nih.gov/nucleotide/226756711?report=genbank&log$=nucltop&blast_rank=7&RID=TACDPN2N01S)  [EB122194](http://www.ncbi.nlm.nih.gov/nucleotide/91011776?report=genbank&log$=nucltop&blast_rank=8&RID=TACDPN2N01S)  [EB125253](http://www.ncbi.nlm.nih.gov/nucleotide/91014835?report=genbank&log$=nucltop&blast_rank=9&RID=TACDPN2N01S)  [EB125340](http://www.ncbi.nlm.nih.gov/nucleotide/91014922?report=genbank&log$=nucltop&blast_rank=11&RID=TACDPN2N01S)  [EB109316](http://www.ncbi.nlm.nih.gov/nucleotide/90998895?report=genbank&log$=nucltop&blast_rank=5&RID=TACDPN2N01S) | At1g62660  (*AtvAINV2*) | 57 | LOC100256970 | 63.7 |
