# Supplementary material for: Expression Patterns of Genes Involved in Sugar Metabolism and Accumulation during Apple Fruit Development
Source: PLoS One. 2012 Mar 7;7(3):e33055. doi: 10.1371/journal.pone.0033055 (PMC3296772; doi:10.1371/journal.pone.0033055)
Supplement: Table S2 — Information of sucrose synthase (SUSY) genes identified in apple. (DOC) [file pone.0033055.s002.doc]

**Table S2** Information of sucrose synthase (SUSY) genes identified in apple

|  | Size  n.t.  (bp)/a.a | *Malus domestica* genome | | *Malus* EST sequence  (Similarity more than 98%) | | Homologous genes | | | |
| --- | --- | --- | --- | --- | --- | --- | --- | --- | --- |
|  |  | Position on Chr | Gene ID |  |  | In *Arabidopsis* | | In *Vitis vinifera* | |
|  |  |  |  |  |  | Locus in TAIR | % similarity (a.a.) | Gene ID in genbank | % similarity (a.a.) |
| *MdSUSY1* | 2424/807 | chr15:15604602..15608264 | MDP0000250070 | [EB133033](http://www.ncbi.nlm.nih.gov/nucleotide/91022615?report=genbank&log$=nucltop&blast_rank=1&RID=N5E36U4G01S)  [CN943285](http://www.ncbi.nlm.nih.gov/nucleotide/48416098?report=genbank&log$=nucltop&blast_rank=2&RID=N5E36U4G01S)  [EB126436](http://www.ncbi.nlm.nih.gov/nucleotide/91016018?report=genbank&log$=nucltop&blast_rank=3&RID=N5E36U4G01S)  [CN891625](http://www.ncbi.nlm.nih.gov/nucleotide/48277867?report=genbank&log$=nucltop&blast_rank=5&RID=N5E36U4G01S)  [EB152271](http://www.ncbi.nlm.nih.gov/nucleotide/91041853?report=genbank&log$=nucltop&blast_rank=6&RID=N5E36U4G01S)  [EB139808](http://www.ncbi.nlm.nih.gov/nucleotide/91029390?report=genbank&log$=nucltop&blast_rank=7&RID=N5E36U4G01S)  [CN898822](http://www.ncbi.nlm.nih.gov/nucleotide/48285063?report=genbank&log$=nucltop&blast_rank=8&RID=N5E36U4G01S)  [CN918916](http://www.ncbi.nlm.nih.gov/nucleotide/48391729?report=genbank&log$=nucltop&blast_rank=9&RID=N5E36U4G01S)  [GO498851](http://www.ncbi.nlm.nih.gov/nucleotide/226741956?report=genbank&log$=nucltop&blast_rank=10&RID=N5E36U4G01S)  [EB139014](http://www.ncbi.nlm.nih.gov/nucleotide/91028596?report=genbank&log$=nucltop&blast_rank=11&RID=N5E36U4G01S) | [CV628760](http://www.ncbi.nlm.nih.gov/nucleotide/54621624?report=genbank&log$=nucltop&blast_rank=12&RID=N5E36U4G01S)  [EB149146](http://www.ncbi.nlm.nih.gov/nucleotide/91038728?report=genbank&log$=nucltop&blast_rank=13&RID=N5E36U4G01S)  [GO515372](http://www.ncbi.nlm.nih.gov/nucleotide/226759426?report=genbank&log$=nucltop&blast_rank=14&RID=N5E36U4G01S)  [CV524378](http://www.ncbi.nlm.nih.gov/nucleotide/53858052?report=genbank&log$=nucltop&blast_rank=15&RID=N5E36U4G01S)  [GO507804](http://www.ncbi.nlm.nih.gov/nucleotide/226750501?report=genbank&log$=nucltop&blast_rank=16&RID=N5E36U4G01S)  [EB140459](http://www.ncbi.nlm.nih.gov/nucleotide/91030041?report=genbank&log$=nucltop&blast_rank=17&RID=N5E36U4G01S)  [GO497995](http://www.ncbi.nlm.nih.gov/nucleotide/226743334?report=genbank&log$=nucltop&blast_rank=18&RID=N5E36U4G01S)  [EB139149](http://www.ncbi.nlm.nih.gov/nucleotide/91028731?report=genbank&log$=nucltop&blast_rank=19&RID=N5E36U4G01S)  [EB132126](http://www.ncbi.nlm.nih.gov/nucleotide/91021708?report=genbank&log$=nucltop&blast_rank=20&RID=N5E36U4G01S) | [At5g20830](http://www.arabidopsis.org/servlets/TairObject?type=locus&name=AT5G20830)  (*AtSUSY1*) | 81.6 | LOC[100249279](http://www.ncbi.nlm.nih.gov/sites/entrez?db=gene&cmd=Retrieve&dopt=full_report&list_uids=100249279) | 81.9 |
| *MdSUSY2* | 2403/800 | chr13:14833784..14838408 | MDP0000293214 | [CN890018](http://www.ncbi.nlm.nih.gov/nucleotide/48276260?report=genbank&log$=nucltop&blast_rank=2&RID=W8U7B75101N) |  | [At5g49190](http://www.arabidopsis.org/servlets/TairObject?type=locus&name=AT5G49190)  (*AtSUSY2*) | 78.7 | LOC[100243135](http://www.ncbi.nlm.nih.gov/sites/entrez?db=gene&cmd=Retrieve&dopt=full_report&list_uids=100243135) | 86.0 |
| *MdSUSY3* | 2439/802 | chr11:34493523..34498858 | MDP0000034697 | [GO501971](http://www.ncbi.nlm.nih.gov/nucleotide/226746715?report=genbank&log$=nucltop&blast_rank=1&RID=N405DCMK01N)  [GO543274](http://www.ncbi.nlm.nih.gov/nucleotide/226787258?report=genbank&log$=nucltop&blast_rank=2&RID=N405DCMK01N)  [DT042332](http://www.ncbi.nlm.nih.gov/nucleotide/71923099?report=genbank&log$=nucltop&blast_rank=3&RID=N405DCMK01N)  [EB122984](http://www.ncbi.nlm.nih.gov/nucleotide/91012566?report=genbank&log$=nucltop&blast_rank=4&RID=N405DCMK01N)  [EB124401](http://www.ncbi.nlm.nih.gov/nucleotide/91013983?report=genbank&log$=nucltop&blast_rank=5&RID=N405DCMK01N)  [EB144194](http://www.ncbi.nlm.nih.gov/nucleotide/91033776?report=genbank&log$=nucltop&blast_rank=6&RID=N405DCMK01N)  [CN912311](http://www.ncbi.nlm.nih.gov/nucleotide/48384811?report=genbank&log$=nucltop&blast_rank=7&RID=N405DCMK01N)  [CN886934](http://www.ncbi.nlm.nih.gov/nucleotide/48273176?report=genbank&log$=nucltop&blast_rank=8&RID=N405DCMK01N)  [EB154567](http://www.ncbi.nlm.nih.gov/nucleotide/91044149?report=genbank&log$=nucltop&blast_rank=9&RID=N405DCMK01N)  [GO529291](http://www.ncbi.nlm.nih.gov/nucleotide/226771533?report=genbank&log$=nucltop&blast_rank=10&RID=N405DCMK01N) | [CO723633](http://www.ncbi.nlm.nih.gov/nucleotide/50703866?report=genbank&log$=nucltop&blast_rank=11&RID=N405DCMK01N)  [CN947570](http://www.ncbi.nlm.nih.gov/nucleotide/48420383?report=genbank&log$=nucltop&blast_rank=13&RID=N405DCMK01N)  [CN883918](http://www.ncbi.nlm.nih.gov/nucleotide/48270160?report=genbank&log$=nucltop&blast_rank=14&RID=N405DCMK01N)  [EB141525](http://www.ncbi.nlm.nih.gov/nucleotide/91031107?report=genbank&log$=nucltop&blast_rank=15&RID=N405DCMK01N)  [EB110991](http://www.ncbi.nlm.nih.gov/nucleotide/91000570?report=genbank&log$=nucltop&blast_rank=16&RID=N405DCMK01N)  [EB110374](http://www.ncbi.nlm.nih.gov/nucleotide/90999953?report=genbank&log$=nucltop&blast_rank=17&RID=N405DCMK01N)  [CN579948](http://www.ncbi.nlm.nih.gov/nucleotide/46991498?report=genbank&log$=nucltop&blast_rank=18&RID=N405DCMK01N)  [EB119609](http://www.ncbi.nlm.nih.gov/nucleotide/91009191?report=genbank&log$=nucltop&blast_rank=19&RID=N405DCMK01N)  [EB114977](http://www.ncbi.nlm.nih.gov/nucleotide/91004556?report=genbank&log$=nucltop&blast_rank=20&RID=N405DCMK01N) | [At4g02280](http://www.arabidopsis.org/servlets/TairObject?type=locus&name=AT4G02280)  (*AtSUSY3*) | 82.2 | LOC[100267606](http://www.ncbi.nlm.nih.gov/sites/entrez?db=gene&cmd=Retrieve&dopt=full_report&list_uids=100267606) | 82.3 |
| *MdSUSY4* | 2337/778 | chr15:23060583..23064022 | MDP0000132527 | [CN899438](http://www.ncbi.nlm.nih.gov/nucleotide/48285679?report=genbank&log$=nucltop&blast_rank=2&RID=N5EVJ97B016)  [CN916151](http://www.ncbi.nlm.nih.gov/nucleotide/48388651?report=genbank&log$=nucltop&blast_rank=3&RID=N5EVJ97B016)  [EB144827](http://www.ncbi.nlm.nih.gov/nucleotide/91034409?report=genbank&log$=nucltop&blast_rank=4&RID=N5EVJ97B016) | [CN908332](http://www.ncbi.nlm.nih.gov/nucleotide/48380833?report=genbank&log$=nucltop&blast_rank=25&RID=N5EVJ97B016)  [EB155578](http://www.ncbi.nlm.nih.gov/nucleotide/91045160?report=genbank&log$=nucltop&blast_rank=32&RID=N5EVJ97B016)  [EB152464](http://www.ncbi.nlm.nih.gov/nucleotide/91042046?report=genbank&log$=nucltop&blast_rank=40&RID=N5EVJ97B016) | [At5g20830](http://www.arabidopsis.org/servlets/TairObject?type=locus&name=AT5G20830)  (*AtSUSY1*) | 78.2 | LOC[100249279](http://www.ncbi.nlm.nih.gov/sites/entrez?db=gene&cmd=Retrieve&dopt=full_report&list_uids=100249279) | 79.8 |
| *MdSUSY5* | 2266/755 | chr17:23733971..23742702 | MDP0000204870 |  |  | [At5g37180](http://www.arabidopsis.org/servlets/TairObject?type=locus&name=AT5G37180)  (*AtSUSY5*) | 69.5 | LOC[100249279](http://www.ncbi.nlm.nih.gov/sites/entrez?db=gene&cmd=Retrieve&dopt=full_report&list_uids=100249279) | 74.5 |
