# Supplementary material for: Expression Patterns of Genes Involved in Sugar Metabolism and Accumulation during Apple Fruit Development
Source: PLoS One. 2012 Mar 7;7(3):e33055. doi: 10.1371/journal.pone.0033055 (PMC3296772; doi:10.1371/journal.pone.0033055)
Supplement: Table S3 — Information of fructokinase (FK) genes identified in apple. (DOC) [file pone.0033055.s003.doc]

**Table S3** Information of fructokinase (FK) genes identified in apple

|  | Size  n.t.  (bp)/a.a | *Malus domestica* genome | | *Malus* EST sequence  (Similarity more than 98%) | | Homologous genes | | | |
| --- | --- | --- | --- | --- | --- | --- | --- | --- | --- |
|  |  | Position on Chr | Gene ID |  |  | In *Arabidopsis* | | In *Lycopersicon esculentum* | |
|  |  |  |  |  |  | Locus in TAIR | % similarity (a.a.) | Gene ID in Genbank | % similarity (a.a.) |
| *MdFK1* | 1077/358 | chr7:23145228..23148363 | MDP0000173131 | [EB123478](http://www.ncbi.nlm.nih.gov/nucleotide/91013060?report=genbank&log$=nucltop&blast_rank=2&RID=MG4JP4BF01N)  [CN903279](http://www.ncbi.nlm.nih.gov/nucleotide/48289519?report=genbank&log$=nucltop&blast_rank=15&RID=MG4JP4BF01N) | [GO565596](http://www.ncbi.nlm.nih.gov/nucleotide/226812614?report=genbank&log$=nucltop&blast_rank=24&RID=MG4JP4BF01N) | [At5g51830](http://www.ncbi.nlm.nih.gov/gene/835258) | 77.3 | [AAB57733](http://www.ncbi.nlm.nih.gov/protein/2102691)  (*LeFK1*) | 73.5 |
| *MdFK2* | 990/329 | chr0:35891547..35894746 | MDP0000323311 | [EE663659](http://www.ncbi.nlm.nih.gov/nucleotide/113374361?report=genbank&log$=nucltop&blast_rank=1&RID=MEC9ZB8G014)  [EB177916](http://www.ncbi.nlm.nih.gov/nucleotide/91068011?report=genbank&log$=nucltop&blast_rank=3&RID=MEC9ZB8G014)  [EB175369](http://www.ncbi.nlm.nih.gov/nucleotide/91065464?report=genbank&log$=nucltop&blast_rank=4&RID=MEC9ZB8G014)  [EB157426](http://www.ncbi.nlm.nih.gov/nucleotide/91047008?report=genbank&log$=nucltop&blast_rank=5&RID=MEC9ZB8G014)  [EB176491](http://www.ncbi.nlm.nih.gov/nucleotide/91066586?report=genbank&log$=nucltop&blast_rank=7&RID=MEC9ZB8G014)  [CO418894](http://www.ncbi.nlm.nih.gov/nucleotide/49635142?report=genbank&log$=nucltop&blast_rank=8&RID=MEC9ZB8G014)  [EB139113](http://www.ncbi.nlm.nih.gov/nucleotide/91028695?report=genbank&log$=nucltop&blast_rank=9&RID=MEC9ZB8G014) | [EB138810](http://www.ncbi.nlm.nih.gov/nucleotide/91028392?report=genbank&log$=nucltop&blast_rank=10&RID=MEC9ZB8G014)  [EB175346](http://www.ncbi.nlm.nih.gov/nucleotide/91065441?report=genbank&log$=nucltop&blast_rank=12&RID=MEC9ZB8G014)  [EB129524](http://www.ncbi.nlm.nih.gov/nucleotide/91019106?report=genbank&log$=nucltop&blast_rank=13&RID=MEC9ZB8G014)  [EB177997](http://www.ncbi.nlm.nih.gov/nucleotide/91068092?report=genbank&log$=nucltop&blast_rank=26&RID=MEC9ZB8G014)  [GO535209](http://www.ncbi.nlm.nih.gov/nucleotide/226780956?report=genbank&log$=nucltop&blast_rank=28&RID=MEC9ZB8G014)  [EB139791](http://www.ncbi.nlm.nih.gov/nucleotide/91029373?report=genbank&log$=nucltop&blast_rank=31&RID=MEC9ZB8G014) | [At2g31390](http://www.ncbi.nlm.nih.gov/gene/817697) | 80.8 | [AAB57734](http://www.ncbi.nlm.nih.gov/protein/2102693)  (*LeFK2*) | 80.9 |
| *MdFK3* | 1101/366 | chr8:15542947..15548147 | MDP0000309723 | [DR990137](http://www.ncbi.nlm.nih.gov/nucleotide/71812746?report=genbank&log$=nucltop&blast_rank=1&RID=MEDR4R5M014)  [CN943926](http://www.ncbi.nlm.nih.gov/nucleotide/48416739?report=genbank&log$=nucltop&blast_rank=2&RID=MEDR4R5M014)  [CN946635](http://www.ncbi.nlm.nih.gov/nucleotide/48419448?report=genbank&log$=nucltop&blast_rank=6&RID=MEDR4R5M014)  [GO527986](http://www.ncbi.nlm.nih.gov/nucleotide/226774037?report=genbank&log$=nucltop&blast_rank=16&RID=MEDR4R5M014)  [CV656732](http://www.ncbi.nlm.nih.gov/nucleotide/54683698?report=genbank&log$=nucltop&blast_rank=31&RID=MEDR4R5M014) | [GO527986](http://www.ncbi.nlm.nih.gov/nucleotide/226774037?report=genbank&log$=nucltop&blast_rank=16&RID=MEDR4R5M014)  [CV656732](http://www.ncbi.nlm.nih.gov/nucleotide/54683698?report=genbank&log$=nucltop&blast_rank=31&RID=MEDR4R5M014)  [CV631128](http://www.ncbi.nlm.nih.gov/nucleotide/54623992?report=genbank&log$=nucltop&blast_rank=33&RID=MEDR4R5M014)  [CV629335](http://www.ncbi.nlm.nih.gov/nucleotide/54622199?report=genbank&log$=nucltop&blast_rank=34&RID=MEDR4R5M014) | [At1g66430](http://www.arabidopsis.org/servlets/TairObject?type=locus&name=AT1G66430) | 76.3 | [AAR2491](http://www.ncbi.nlm.nih.gov/protein/38604456)  (*LeFK3*) | 79.3 |
| *MdFK4* | 1077/358 | chr1:24968994..24972364 | MDP0000765663 | [CN899660](http://www.ncbi.nlm.nih.gov/nucleotide/48285901?report=genbank&log$=nucltop&blast_rank=1&RID=M9FW4KXJ016)  [CN941706](http://www.ncbi.nlm.nih.gov/nucleotide/48414519?report=genbank&log$=nucltop&blast_rank=2&RID=M9FW4KXJ016)  [EB146470](http://www.ncbi.nlm.nih.gov/nucleotide/91036052?report=genbank&log$=nucltop&blast_rank=3&RID=M9FW4KXJ016)  [GO510279](http://www.ncbi.nlm.nih.gov/nucleotide/226755269?report=genbank&log$=nucltop&blast_rank=4&RID=M9FW4KXJ016) | [DT002797](http://www.ncbi.nlm.nih.gov/nucleotide/71825405?report=genbank&log$=nucltop&blast_rank=5&RID=M9FW4KXJ016)  [CN491344](http://www.ncbi.nlm.nih.gov/nucleotide/46607986?report=genbank&log$=nucltop&blast_rank=18&RID=M9FW4KXJ016)  [CN877754](http://www.ncbi.nlm.nih.gov/nucleotide/48263994?report=genbank&log$=nucltop&blast_rank=23&RID=M9FW4KXJ016) | [At5g51830](http://www.ncbi.nlm.nih.gov/gene/835258) | 76.7 | [AAM44084](http://www.ncbi.nlm.nih.gov/protein/23476263)  (*LeFK4*) | 72.4 |
