# Supplementary material for: Expression Patterns of Genes Involved in Sugar Metabolism and Accumulation during Apple Fruit Development
Source: PLoS One. 2012 Mar 7;7(3):e33055. doi: 10.1371/journal.pone.0033055 (PMC3296772; doi:10.1371/journal.pone.0033055)
Supplement: Table S4 — Information of hexokinase (HK) genes identified in apple. (DOC) [file pone.0033055.s004.doc]

**Table S4** Information of hexokinase (HK) genes identified in apple

|  | Size  n.t.  (bp)/a.a | *Malus domestica* genome | | *Malus* EST sequence  (Similarity more than 98%) | | Homologous genes | | | |
| --- | --- | --- | --- | --- | --- | --- | --- | --- | --- |
|  |  | Position on Chr | Gene ID |  |  | In *Arabidopsis* | | In *Vitis vinifera* | |
|  |  |  |  |  |  | Locus in TAIR | % similarity (a.a.) | Gene ID in Genbank | % similarity (a.a.) |
| *MdHK1* | 1497/498 | chr15:13844251..13848010 | MDP0000309677 | [CO904487](http://www.ncbi.nlm.nih.gov/nucleotide/51294790?report=genbank&log$=nucltop&blast_rank=3&RID=UYD1GYXX015)  [EB134363](http://www.ncbi.nlm.nih.gov/nucleotide/91023945?report=genbank&log$=nucltop&blast_rank=4&RID=UYD1GYXX015)  [CV082226](http://www.ncbi.nlm.nih.gov/nucleotide/51561029?report=genbank&log$=nucltop&blast_rank=5&RID=UYD1GYXX015)  [EB139114](http://www.ncbi.nlm.nih.gov/nucleotide/91028696?report=genbank&log$=nucltop&blast_rank=6&RID=UYD1GYXX015)  [CN879283](http://www.ncbi.nlm.nih.gov/nucleotide/48265523?report=genbank&log$=nucltop&blast_rank=7&RID=UYD1GYXX015)  [EB128444](http://www.ncbi.nlm.nih.gov/nucleotide/91018026?report=genbank&log$=nucltop&blast_rank=8&RID=UYD1GYXX015)  [CO865432](http://www.ncbi.nlm.nih.gov/nucleotide/51095582?report=genbank&log$=nucltop&blast_rank=12&RID=UYD1GYXX015) | [GO511073](http://www.ncbi.nlm.nih.gov/nucleotide/226755071?report=genbank&log$=nucltop&blast_rank=21&RID=UYD1GYXX015)  [GO518800](http://www.ncbi.nlm.nih.gov/nucleotide/226764003?report=genbank&log$=nucltop&blast_rank=22&RID=UYD1GYXX015)  [CN496753](http://www.ncbi.nlm.nih.gov/nucleotide/46599696?report=genbank&log$=nucltop&blast_rank=26&RID=UYD1GYXX015)  [GO503140](http://www.ncbi.nlm.nih.gov/nucleotide/226748383?report=genbank&log$=nucltop&blast_rank=30&RID=UYD1GYXX015)  [EB135080](http://www.ncbi.nlm.nih.gov/nucleotide/91024662?report=genbank&log$=nucltop&blast_rank=38&RID=UYD1GYXX015)  [CO901116](http://www.ncbi.nlm.nih.gov/nucleotide/51240906?report=genbank&log$=nucltop&blast_rank=68&RID=UYD1GYXX015) | At4g29130  (*AtHK1*) | 77.6 | LOC100242358 | 82.4 |
| *MdHK2* | 1494/497 | chr9:18945732..18949202 | MDP0000823956 | [CN902471](http://www.ncbi.nlm.nih.gov/nucleotide/48288711?report=genbank&log$=nucltop&blast_rank=2&RID=UYF4RKVF011)  [CN579960](http://www.ncbi.nlm.nih.gov/nucleotide/46991510?report=genbank&log$=nucltop&blast_rank=3&RID=UYF4RKVF011)  [CN905457](http://www.ncbi.nlm.nih.gov/nucleotide/48377958?report=genbank&log$=nucltop&blast_rank=4&RID=UYF4RKVF011)  [CN906068](http://www.ncbi.nlm.nih.gov/nucleotide/48378569?report=genbank&log$=nucltop&blast_rank=5&RID=UYF4RKVF011) | [CN905110](http://www.ncbi.nlm.nih.gov/nucleotide/48377611?report=genbank&log$=nucltop&blast_rank=6&RID=UYF4RKVF011)  [CN904306](http://www.ncbi.nlm.nih.gov/nucleotide/48376807?report=genbank&log$=nucltop&blast_rank=7&RID=UYF4RKVF011)  [CN905172](http://www.ncbi.nlm.nih.gov/nucleotide/48377673?report=genbank&log$=nucltop&blast_rank=13&RID=UYF4RKVF011) | At4g29130 (*AtHK1*) | 70.6 | LOC100244595 | 75.3 |
| *MdHK3* | 1479/492 | chr12:21201014..21203429 | MDP0000028419 | [CV129686](http://www.ncbi.nlm.nih.gov/nucleotide/51865205?report=genbank&log$=nucltop&blast_rank=1&RID=UYC5U0K1011)  [DT040183](http://www.ncbi.nlm.nih.gov/nucleotide/71918572?report=genbank&log$=nucltop&blast_rank=2&RID=UYC5U0K1011)  [GO565302](http://www.ncbi.nlm.nih.gov/nucleotide/226812672?report=genbank&log$=nucltop&blast_rank=3&RID=UYC5U0K1011)  [GO550338](http://www.ncbi.nlm.nih.gov/nucleotide/226795125?report=genbank&log$=nucltop&blast_rank=4&RID=UYC5U0K1011) | [CN863464](http://www.ncbi.nlm.nih.gov/nucleotide/48120068?report=genbank&log$=nucltop&blast_rank=5&RID=UYC5U0K1011)  [CN897749](http://www.ncbi.nlm.nih.gov/nucleotide/48283990?report=genbank&log$=nucltop&blast_rank=6&RID=UYC5U0K1011)  [CN940622](http://www.ncbi.nlm.nih.gov/nucleotide/48413435?report=genbank&log$=nucltop&blast_rank=7&RID=UYC5U0K1011)  [CN918171](http://www.ncbi.nlm.nih.gov/nucleotide/48390671?report=genbank&log$=nucltop&blast_rank=15&RID=UYC5U0K1011) | At1g47840  (*AtHK3*) | 63.5 | LOC100255753 | 88.2 |
| *MdHK4* | 1542/513 | chr2:20752837..20755575 | MDP0000272613 |  |  | [At4g37840](http://www.arabidopsis.org/servlets/TairObject?type=locus&name=AT4G37840)  (*AtHKL3*) | 53.1 | LOC100262786 | 59.3 |
| *MdHK5* | 1557/518 | chr3:20898986..20904663 | MDP0000128035 | [CO416154](http://www.ncbi.nlm.nih.gov/nucleotide/49632402?report=genbank&log$=nucltop&blast_rank=2&RID=UYES96G2015)  [GO504002](http://www.ncbi.nlm.nih.gov/nucleotide/226748161?report=genbank&log$=nucltop&blast_rank=3&RID=UYES96G2015) | [CN444979](http://www.ncbi.nlm.nih.gov/nucleotide/46424243?report=genbank&log$=nucltop&blast_rank=10&RID=UYES96G2015) | At1g50460  (*AtHKL1*) | 67.9 | LOC100263580 | 76.7 |
