# Supplementary material for: Expression Patterns of Genes Involved in Sugar Metabolism and Accumulation during Apple Fruit Development
Source: PLoS One. 2012 Mar 7;7(3):e33055. doi: 10.1371/journal.pone.0033055 (PMC3296772; doi:10.1371/journal.pone.0033055)
Supplement: Table S5 — Information of sucrose-phosphate synthase (SPS) genes identified in apple. (DOC) [file pone.0033055.s005.doc]

**Table S5** Information of sucrose-phosphate synthase (SPS) genes identified in apple

|  | Size  n.t.  (bp)/a.a | *Malus domestica* genome | | *Malus* EST sequence  (Similarity more than 98%) | | Homologous genes | | | |
| --- | --- | --- | --- | --- | --- | --- | --- | --- | --- |
|  |  | Position on Chr | Gene ID |  |  | In *Arabidopsis* | | In *Vitis vinifera* | |
|  |  |  |  |  |  | Locus in TAIR | % similarity (a.a.) | Gene ID in Genbank | % similarity (a.a.) |
| *MdSPS1* | 3168/1055 | chr2:2524387..2529864 | MDP0000855287 | [DT002030](http://www.ncbi.nlm.nih.gov/nucleotide/71824638?report=genbank&log$=nucltop&blast_rank=1&RID=N3V4ZZ6P014)  [EB138494](http://www.ncbi.nlm.nih.gov/nucleotide/91028076?report=genbank&log$=nucltop&blast_rank=2&RID=N3V4ZZ6P014)  [CV880703](http://www.ncbi.nlm.nih.gov/nucleotide/55855911?report=genbank&log$=nucltop&blast_rank=3&RID=N3V4ZZ6P014)  [CV085786](http://www.ncbi.nlm.nih.gov/nucleotide/51565135?report=genbank&log$=nucltop&blast_rank=4&RID=N3V4ZZ6P014)  [EB125809](http://www.ncbi.nlm.nih.gov/nucleotide/91015391?report=genbank&log$=nucltop&blast_rank=5&RID=N3V4ZZ6P014)  [EB155087](http://www.ncbi.nlm.nih.gov/nucleotide/91044669?report=genbank&log$=nucltop&blast_rank=6&RID=N3V4ZZ6P014)  [EB140696](http://www.ncbi.nlm.nih.gov/nucleotide/91030278?report=genbank&log$=nucltop&blast_rank=7&RID=N3V4ZZ6P014)  [EB142546](http://www.ncbi.nlm.nih.gov/nucleotide/91032128?report=genbank&log$=nucltop&blast_rank=9&RID=N3V4ZZ6P014) | [EB125438](http://www.ncbi.nlm.nih.gov/nucleotide/91015020?report=genbank&log$=nucltop&blast_rank=19&RID=N3V4ZZ6P014)  [CV997459](http://www.ncbi.nlm.nih.gov/nucleotide/56200494?report=genbank&log$=nucltop&blast_rank=20&RID=N3V4ZZ6P014)  [CN444838](http://www.ncbi.nlm.nih.gov/nucleotide/46424102?report=genbank&log$=nucltop&blast_rank=21&RID=N3V4ZZ6P014)  [GO527077](http://www.ncbi.nlm.nih.gov/nucleotide/226772162?report=genbank&log$=nucltop&blast_rank=26&RID=N3V4ZZ6P014)  [EB122136](http://www.ncbi.nlm.nih.gov/nucleotide/91011718?report=genbank&log$=nucltop&blast_rank=29&RID=N3V4ZZ6P014)  [EB115310](http://www.ncbi.nlm.nih.gov/nucleotide/91004889?report=genbank&log$=nucltop&blast_rank=35&RID=N3V4ZZ6P014)  [EB112628](http://www.ncbi.nlm.nih.gov/nucleotide/91002207?report=genbank&log$=nucltop&blast_rank=37&RID=N3V4ZZ6P014)  [CN544859](http://www.ncbi.nlm.nih.gov/nucleotide/46873015?report=genbank&log$=nucltop&blast_rank=39&RID=N3V4ZZ6P014) | At5g20280  (*AtSPSA1*) | 77.4 | LOC100241955 | 88.2 |
| *MdSPS2* | 3195/1064 | chr15:9106119..9112095 | MDP0000213919 | [EB140478](http://www.ncbi.nlm.nih.gov/nucleotide/91030060?report=genbank&log$=nucltop&blast_rank=1&RID=N3VTBKTR01N)  [CO417339](http://www.ncbi.nlm.nih.gov/nucleotide/49633587?report=genbank&log$=nucltop&blast_rank=8&RID=N3VTBKTR01N)  [GO499277](http://www.ncbi.nlm.nih.gov/nucleotide/226743249?report=genbank&log$=nucltop&blast_rank=12&RID=N3VTBKTR01N) | [GO508909](http://www.ncbi.nlm.nih.gov/nucleotide/226753254?report=genbank&log$=nucltop&blast_rank=16&RID=N3VTBKTR01N)  [EB137092](http://www.ncbi.nlm.nih.gov/nucleotide/91026674?report=genbank&log$=nucltop&blast_rank=20&RID=N3VTBKTR01N) | At5g20280  (*AtSPSA1*) | 77.1 | LOC100241955 | 82.4 |
| *MdSPS3* | 3177/1058 | chr15:6181969..6187418 | MDP0000414968 | [EB145518](http://www.ncbi.nlm.nih.gov/nucleotide/91035100?report=genbank&log$=nucltop&blast_rank=1&RID=N3XEDNV2014)  [EB123469](http://www.ncbi.nlm.nih.gov/nucleotide/91013051?report=genbank&log$=nucltop&blast_rank=2&RID=N3XEDNV2014)  [EB133957](http://www.ncbi.nlm.nih.gov/nucleotide/91023539?report=genbank&log$=nucltop&blast_rank=3&RID=N3XEDNV2014)  [EB155170](http://www.ncbi.nlm.nih.gov/nucleotide/91044752?report=genbank&log$=nucltop&blast_rank=4&RID=N3XEDNV2014) | [GO513969](http://www.ncbi.nlm.nih.gov/nucleotide/226759451?report=genbank&log$=nucltop&blast_rank=7&RID=N3XEDNV2014)  [EB123671](http://www.ncbi.nlm.nih.gov/nucleotide/91013253?report=genbank&log$=nucltop&blast_rank=20&RID=N3XEDNV2014)  [GO547701](http://www.ncbi.nlm.nih.gov/nucleotide/226791808?report=genbank&log$=nucltop&blast_rank=22&RID=N3XEDNV2014) | [At5g11110](http://www.arabidopsis.org/servlets/TairObject?type=locus&name=AT5G11110)  (*AtSPSA2*) | 71.7 | LOC100244135 | 76.7 |
| *MdSPS4* | 3198/1065 | chr9:15144861..15149936 | MDP0000783676 | [CN495376](http://www.ncbi.nlm.nih.gov/nucleotide/46596570?report=genbank&log$=nucltop&blast_rank=3&RID=N3WDSXDZ012)  [GO514123](http://www.ncbi.nlm.nih.gov/nucleotide/226757890?report=genbank&log$=nucltop&blast_rank=7&RID=N3WDSXDZ012)  [GO547689](http://www.ncbi.nlm.nih.gov/nucleotide/226791770?report=genbank&log$=nucltop&blast_rank=8&RID=N3WDSXDZ012) | [EB134384](http://www.ncbi.nlm.nih.gov/nucleotide/91023966?report=genbank&log$=nucltop&blast_rank=9&RID=N3WDSXDZ012)  [CO068829](http://www.ncbi.nlm.nih.gov/nucleotide/48738310?report=genbank&log$=nucltop&blast_rank=10&RID=N3WDSXDZ012)  [CN902363](http://www.ncbi.nlm.nih.gov/nucleotide/48288603?report=genbank&log$=nucltop&blast_rank=11&RID=N3WDSXDZ012) | At1g04920  (*AtSPSB*) | 76.0 | LOC100262385 | 82.6 |
| *MdSPS5* | 3042/1013 | chr10:319926..325511 | MDP0000147573 | [CV084273](http://www.ncbi.nlm.nih.gov/nucleotide/51563534?report=genbank&log$=nucltop&blast_rank=1&RID=N3YPATNS01S) | [GO567037](http://www.ncbi.nlm.nih.gov/nucleotide/226812485?report=genbank&log$=nucltop&blast_rank=43&RID=N3YPATNS01S) | At4g10120  (*AtSPSC*) | 68.9 | LOC100232974  (*VvSPS1*) | 75.3 |
| *MdSPS6* | 3075/1024 | chr10:298880..304254 | MDP0000288684 | [EB140964](http://www.ncbi.nlm.nih.gov/nucleotide/91030546?report=genbank&log$=nucltop&blast_rank=1&RID=N419Z21201N)  [CN911211](http://www.ncbi.nlm.nih.gov/nucleotide/48383712?report=genbank&log$=nucltop&blast_rank=2&RID=N419Z21201N)  [GO504939](http://www.ncbi.nlm.nih.gov/nucleotide/226749817?report=genbank&log$=nucltop&blast_rank=3&RID=N419Z21201N)  [CV883173](http://www.ncbi.nlm.nih.gov/nucleotide/55858381?report=genbank&log$=nucltop&blast_rank=4&RID=N419Z21201N)  [EB154526](http://www.ncbi.nlm.nih.gov/nucleotide/91044108?report=genbank&log$=nucltop&blast_rank=5&RID=N419Z21201N)  [CO868159](http://www.ncbi.nlm.nih.gov/nucleotide/51098309?report=genbank&log$=nucltop&blast_rank=9&RID=N419Z21201N)  [GO522294](http://www.ncbi.nlm.nih.gov/nucleotide/226767709?report=genbank&log$=nucltop&blast_rank=23&RID=N419Z21201N) | [EB117788](http://www.ncbi.nlm.nih.gov/nucleotide/91007367?report=genbank&log$=nucltop&blast_rank=10&RID=N419Z21201N)  [CV632125](http://www.ncbi.nlm.nih.gov/nucleotide/54624989?report=genbank&log$=nucltop&blast_rank=17&RID=N419Z21201N)  [EB156915](http://www.ncbi.nlm.nih.gov/nucleotide/91046497?report=genbank&log$=nucltop&blast_rank=18&RID=N419Z21201N)  [CN875118](http://www.ncbi.nlm.nih.gov/nucleotide/48261322?report=genbank&log$=nucltop&blast_rank=19&RID=N419Z21201N)  [CO416417](http://www.ncbi.nlm.nih.gov/nucleotide/49632665?report=genbank&log$=nucltop&blast_rank=20&RID=N419Z21201N)  [EE663925](http://www.ncbi.nlm.nih.gov/nucleotide/113374627?report=genbank&log$=nucltop&blast_rank=22&RID=N419Z21201N)  [GO560472](http://www.ncbi.nlm.nih.gov/nucleotide/226804669?report=genbank&log$=nucltop&blast_rank=25&RID=N419Z21201N) | At4g10120  (*AtSPSC*) | 73.3 | LOC100232974  (*VvSPS1*) | 80.5 |
