# Supplementary material for: Expression Patterns of Genes Involved in Sugar Metabolism and Accumulation during Apple Fruit Development
Source: PLoS One. 2012 Mar 7;7(3):e33055. doi: 10.1371/journal.pone.0033055 (PMC3296772; doi:10.1371/journal.pone.0033055)
Supplement: Table S6 — Information of sucrose transporter (SUT), tonoplast monosaccharide transporter (TMT) and vacuole glucose transporter (vGT) genes identified in apple. (DOC) [file pone.0033055.s006.doc]

**Table S6** Information of sucrose transporter (SUT), tonoplast monosaccharide transporter (TMT) and vacuole glucose transporter (vGT) genes identified in apple

|  | Size  n.t.  (bp)/a.a | *Malus domestica* genome | | *Malus* EST sequence  (Similarity more than 98%) | | Homologous genes | | | |
| --- | --- | --- | --- | --- | --- | --- | --- | --- | --- |
|  |  | Position on Chr | Gene ID |  |  | In *Arabidopsis* | | In *Vitis vinifera* | |
|  |  |  |  |  |  | Locus in TAIR | % similarity (a.a.) | Gene ID in genbank | % similarity (a.a.) |
| *MdSUT1* | 1500/499 | chr15:39745672..39749711 | MDP0000275743 | [DT042011](http://www.ncbi.nlm.nih.gov/nucleotide/71922409?report=genbank&log$=nucltop&blast_rank=1&RID=UHMTF951011)  [EB144622](http://www.ncbi.nlm.nih.gov/nucleotide/91034204?report=genbank&log$=nucltop&blast_rank=3&RID=UHMTF951011) | [EB145181](http://www.ncbi.nlm.nih.gov/nucleotide/91034763?report=genbank&log$=nucltop&blast_rank=2&RID=UHMTF951011) | At1g09960  (*AtSUT4*) | 70.8 | LOC[100232844](http://www.ncbi.nlm.nih.gov/entrez/utils/fref.fcgi?itool=Gene_FullReport&PrId=5174&uid=100232844&nlmid=&db=gene&url=http://www.genome.jp/dbget-bin/www_bget?vvi:100232844)  (*VvSUT11*) | 76 |
| *MdSUT2* | 1899/632 | chr13:4786129..4791103 | MDP0000850943 | [DT000487](http://www.ncbi.nlm.nih.gov/nucleotide/71823095?report=genbank&log$=nucltop&blast_rank=2&RID=UHPWF9ZE011) |  | At2g02860  (*AtSUT3*) | 72.6 | LOC[100232845](http://www.ncbi.nlm.nih.gov/entrez/utils/fref.fcgi?itool=Gene_FullReport&PrId=5174&uid=100232845&nlmid=&db=gene&url=http://www.genome.jp/dbget-bin/www_bget?vvi:100232845)  (*VvSUT12*) | 75.3 |
| *MdSUT3* | 1530/509 | chr8:25356318..25360092 | MDP0000206996 |  |  | At1g09960  (*AtSUT4*) | 68.6 | LOC[100232844](http://www.ncbi.nlm.nih.gov/entrez/utils/fref.fcgi?itool=Gene_FullReport&PrId=5174&uid=100232844&nlmid=&db=gene&url=http://www.genome.jp/dbget-bin/www_bget?vvi:100232844)  (*VvSUT11*) | 74.3 |
| *MdSUT4* | 1263/420 | chr10:5601395..5603062 | MDP0000897552 | [GO504094](http://www.ncbi.nlm.nih.gov/nucleotide/226749349?report=genbank&log$=nucltop&blast_rank=1&RID=XBGFFKE9014)  [EB132889](http://www.ncbi.nlm.nih.gov/nucleotide/91022471?report=genbank&log$=nucltop&blast_rank=2&RID=XBGFFKE9014) | [EB128361](http://www.ncbi.nlm.nih.gov/nucleotide/91017943?report=genbank&log$=nucltop&blast_rank=3&RID=XBGFFKE9014)  [GO508400](http://www.ncbi.nlm.nih.gov/nucleotide/226750589?report=genbank&log$=nucltop&blast_rank=4&RID=XBGFFKE9014) | [At5g43610](http://www.arabidopsis.org/servlets/TairObject?type=locus&name=AT5G43610)  (*AtSUT6*) | 62.0 | LOC[100232846](http://www.ncbi.nlm.nih.gov/entrez/utils/fref.fcgi?itool=Gene_FullReport&PrId=5174&uid=100232846&nlmid=&db=gene&url=http://www.genome.jp/dbget-bin/www_bget?vvi:100232846)  (*VvSUT27*) | 67.7 |
| *MdSUT5* | 1467/488 | chr9:22390027..22406352 | MDP0000426862 |  |  | At1g71880 (*AtSUT1*) | 64.8 | LOC[100232846](http://www.ncbi.nlm.nih.gov/entrez/utils/fref.fcgi?itool=Gene_FullReport&PrId=5174&uid=100232846&nlmid=&db=gene&url=http://www.genome.jp/dbget-bin/www_bget?vvi:100232846)  (*VvSUT27*) | 67.3 |
| *MdTMT1* | 2220/739 | chr6:776756..781020 | MDP0000381084 | [CN941160](http://www.ncbi.nlm.nih.gov/nucleotide/48413973?report=genbank&log$=nucltop&blast_rank=3&RID=XXC0FX8G014)  [CN874916](http://www.ncbi.nlm.nih.gov/nucleotide/48261104?report=genbank&log$=nucltop&blast_rank=4&RID=XXC0FX8G014)  [GO531462](http://www.ncbi.nlm.nih.gov/nucleotide/226774210?report=genbank&log$=nucltop&blast_rank=5&RID=XXC0FX8G014)  [GO522072](http://www.ncbi.nlm.nih.gov/nucleotide/226765644?report=genbank&log$=nucltop&blast_rank=6&RID=XXC0FX8G014)  [CN890611](http://www.ncbi.nlm.nih.gov/nucleotide/48276853?report=genbank&log$=nucltop&blast_rank=7&RID=XXC0FX8G014)  [GO500899](http://www.ncbi.nlm.nih.gov/nucleotide/226747018?report=genbank&log$=nucltop&blast_rank=9&RID=XXC0FX8G014) | [CN995112](http://www.ncbi.nlm.nih.gov/nucleotide/48487002?report=genbank&log$=nucltop&blast_rank=21&RID=XXC0FX8G014)  [CV092117](http://www.ncbi.nlm.nih.gov/nucleotide/51571456?report=genbank&log$=nucltop&blast_rank=29&RID=XXC0FX8G014)  [CN926458](http://www.ncbi.nlm.nih.gov/nucleotide/48399271?report=genbank&log$=nucltop&blast_rank=47&RID=XXC0FX8G014)  [CN860578](http://www.ncbi.nlm.nih.gov/nucleotide/48116741?report=genbank&log$=nucltop&blast_rank=58&RID=XXC0FX8G014)  [CN931875](http://www.ncbi.nlm.nih.gov/nucleotide/48404688?report=genbank&log$=nucltop&blast_rank=60&RID=XXC0FX8G014) | At1g20840 (*AtTMT1*) | 71.0 | LOC100232977  (*VvHT6* or *VvTMT1*) | 80.8 |
| *MdTMT2* | 2211/736 | chr8:10037709..10040893 | MDP0000868028 | [DR997752](http://www.ncbi.nlm.nih.gov/nucleotide/71820361?report=genbank&log$=nucltop&blast_rank=3&RID=XXCRR9XJ016) | [GO524570](http://www.ncbi.nlm.nih.gov/nucleotide/226768964?report=genbank&log$=nucltop&blast_rank=10&RID=XXCRR9XJ016) | At4g35300 (*AtTMT2*) | 75.0 | LOC100264011 (*VvTMT2*) | 76.9 |
| *MdTMT3* | 2211/736 | chr10:13737422..13740641 | MDP0000212510 | [CN899897](http://www.ncbi.nlm.nih.gov/nucleotide/48286138?report=genbank&log$=nucltop&blast_rank=3&RID=XXD7F08R014)  CV658131 | [GO535083](http://www.ncbi.nlm.nih.gov/nucleotide/226779266?report=genbank&log$=nucltop&blast_rank=6&RID=XXD7F08R014)  [CV881666](http://www.ncbi.nlm.nih.gov/nucleotide/55856874?report=genbank&log$=nucltop&blast_rank=7&RID=XXD7F08R014) | At4g35300 (*AtTMT2*) | 75.2 | LOC100264011 (*VvTMT2*) | 75.3 |
| MdTMT4 | 2080/759 | [chr9:7632993..7635873](http://www.rosaceae.org/gb/gbrowse/malus_x_domestica?name=chr9:7632993..7635873) | MDP0000007886 | [GO509761](http://www.ncbi.nlm.nih.gov/nucleotide/226756825?report=genbank&log$=nucltop&blast_rank=1&RID=XXDW6CJR014) |  | At3g51490 (*AtTMT3*) | 63.1 | LOC[100243856](http://www.ncbi.nlm.nih.gov/sites/entrez?db=gene&cmd=Retrieve&dopt=full_report&list_uids=100243856)  (*VvTMT3*) | 71.7 |
| MdTMT5 | 2211/736 | chr9:7637075..7639966 | MDP0000872215 |  |  | At3g51490 (*AtTMT3*) | 58.9 | LOC[100243856](http://www.ncbi.nlm.nih.gov/sites/entrez?db=gene&cmd=Retrieve&dopt=full_report&list_uids=100243856) (*VvTMT3*) | 64.8 |
| *MdVGT1* | 1506/501 | chr1:29137621..29141041 | MDP0000863082 | CN996796 |  | At5g17010  (*AtvGT2*) | 75.8 | LOC100258018 | 80.3 |
| *MdvGT2* | 1656/551 | chr7:26339899..26343418 | MDP0000150156 | CV092141 |  | At5g17010  (*AtvGT2*) | 75.7 | LOC100258018 | 78.5 |
