# Supplementary material for: Expression Patterns of Genes Involved in Sugar Metabolism and Accumulation during Apple Fruit Development
Source: PLoS One. 2012 Mar 7;7(3):e33055. doi: 10.1371/journal.pone.0033055 (PMC3296772; doi:10.1371/journal.pone.0033055)
Supplement: Table S7 — Oligonucleotide sequences for primers used in this study. (DOC) [file pone.0033055.s007.doc]

**Table S7** Oligonucleotide sequences for primers used in this study

| Gene | Sense (5’-3’) | Anti-sense (5’-3’) |
| --- | --- | --- |
| *MdSDH1* | ATA GAG GAA GTT GGG AGT GAG GT | TCT CCT GGA TGG ACA ACC TGA TT |
| *MdSDH2-9* | ACA CCA TCA AGA TCC TAC CTT TC | CAT TTC ATG GTC TTG AGG TAG TG |
| *MdCWIN1* | TAA CAA ATA TGT GGT GCT CCT CTG | ACC CTA GCT GTT ATG CAC GCC T |
| *MdCWINV2* | TTC AAA GCT AAA GGC AGA CAC G | GTA AAT CTA CAT CTA CAA AGC CAG C |
| *MdCWINV3* | TAG ACC ATC ATT TGC TGG CTA TGT | TAA GGT GGG ATA AAC CCT GGA TA |
| *MdNINV1* | GTC CAT TGT TTC ATC ATT GGG TAC | GGT CGC TGC CAG TGA TTA TAC G |
| *MdNINV2* | GAG TTC CAG ACA GGC ATA AGG CT | CCA TCC GTC TAT CAA TCA TAC AGG |
| *MdNINV3* | GGT ACT TGG TAG CGA AGA TGA TGT | ACC AAA CCC TTG TGC CGA TTA |
| *MdvAINV1* | AGG TCA AGG CAG GCT CAG TGC T | CCT AAA GCA CCA CGA TGG GAA GC |
| *MdvAINV2* | ACA GAA GCA ATC TAT GGA GCA GC | GTA AAT CTG GTC TAA TGG GAA AGG |
| *MdvAINV3* | TCA AAC AAG TTT ATG GAG GCG AAG | ACC TGG TCT AAT GGG AAT GGA TG |
| *MdSUSY1* | CTC AAG CGT GTT AAG CAA CAG | CTG AAT GGA ACA CGA AGA ATA TC |
| *MdSUSY2* | TGT GGT TGG TGG TTA CAT GGA TG | GCT GCT ATC CAT CGG AAC TGA C |
| *MdSUSY3* | TTA TGG TTT CTG GAA GTA TGT GTC | GTC GAT GGC TTC AGG AAC AGA TT |
| *MdSUSY4* | GAC AGG AAC AAG CCA ATC ATC T | GCC TTC TCC TCA TTG TCC TTG |
| *MdSUSY5* | CAT GCC AAT TTC TTG CTG ACA C | CTT TCG TAT TGT CCT GGT CTG TC |
| *MdFK1* | CTG CAT TGG CAT TTG TTA CAC TC | AAG ATG GGT TGA CCT GCA TGG T |
| *MdFK2* | GTG GTG GAA TCC TTC GAG GTC AA | CAA ATT TCA GTA CCT CCC TCA ACC T |
| *MdFK3* | AGA GTC AAG GGT ATG AAG GTA GAT G | CTC GTC CTG AAG CAA AGA AAG AT |
| *MdFK4* | TCA GGA TGA GGA GGG GCT ACG AG | CTG CTT TAA GCA CTG GAG CAC AGC |
| *MdHK1* | CTG AAA GTG GTC GGG AGC AAA C | TGC ACG AGT GGC AAC TAT GTC G |
| *MdHK2* | TGG TGG ATT ATA CGA GCA TTA CA | TCC AGG GTA TTG TGA GTG AGA G |
| *MdHK3* | AGA TTG TGG CGG ATG TAT GTG AC | CAA CAG TCC TCT TGC CAA AAA TG |
| *MdHK4* | GAT CAT GAC CCT GAT ACT GAT CCA G | CGG ATT AGG ATC ACG AAC ATC AT |
| *MdHK5* | AGT GGG GAA GAG TGT TTG GGG TC | CCA CCG TCA GAG GCC AAG CCA |
| *MdHK6* | GTG GGG CAG AGT GTT TGG TGT T | AAC CAC CGT CAG AGG CCA AAC C |
| *MdSPS1* | AGT GTA GTA CTC AAG GGA GTT GG | TGC TCA TGG GGA AGG CTT TAC |
| *MdSPS2* | GTG TAG TGC TGA AAG GAG TTG C | CTA GTT TCT CCA AGG AAG CAC G |
| *MdSPS3* | TCA TTG AGA AGG TTA CTG AGG CAG | ACC ACC ACT GTT ACA GAT AAA AGC |
| *MdSPS4* | GCT ATG CCA ATG TTA GAA ACT GTA G | ACA ACC CCA ACG GTA GTC AAT AT |
| *MdSPS5* | TGT TAC TGC CCA TGA TTC CGA | TGC AAG TAT AGT GGG CTT GTG AG |
| *MdSPS6* | AGG TTC TGT TGA GTA TGG CAG TGA G | GTG CTT CAA GTG CCG CTG AGA |
| *MdSOT1* | GGT TAG AAT GAC GTG GGC AGT TAT | TCA TCA ACC TAT TCA CGG CCA C |
| *MdSOT2* | ACA AGG GTC AAG TCC ACT AGG CA | CCG ATA AGA TGT AAT TGG CAA CAG |
| *MdSUT1* | TGT TCC GTA TGC TTT GGT TTC TTC | AAT AGC TGA TCC CAA GGT CCA CT |
| *MdSUT2* | ACT CAC TAT GTA TCA GCA GAA T | TGA GAT GGC CTC CTT TAG ATT CT |
| *MdSUT3* | GTG AAC AAA AGT ATT GGC ATT TC | TCG AGA AGA AAC CAG AGC ATA T |
| *MdSUT4* | CCA ACA AGA ATC CTC ACG CCG | GGT GAG GAG GGA GAG TTG TAG G |
| *MdSUT5* | CCG ACA AGA ATT CTC AGG CCA | GGT CAG GAG GGA GAG TTG CAG T |
| *MdTMT1* | TCG TCT ATT TCT GCG TCT TTG TC | CCG CTG CGT AAA TCC CAA AT |
| *MdTMT2* | GTA CCG AAC GAT GGT CAG TTC TTC | TGA CTC CGG GTT CGA AAA GGT C |
| *MdTMT3* | GCA CCG AAC GAC AGT GAG TTC ATT | GTC ACT CCA GGT TCA AAA AGA TC |
| *MdTMT4* | AGG CCT TAG TTC CAC CTC TTC ATC T | CGG TAA TGT AGT CAG CAA CAG CGA |
| *MdTMT5* | GCG AAG ACG GAA AGA ATG AAG GAG | CCA GAG CAG CAG CAT GAA TGA GTT |
| *MdvGT1* | CTG TTC TTG TTG TTG ATA GGC TCG | ACA TAC AGC AGC AGA GCA ACT ACA |
| *MdvGT2* | TCT TGT TGT TGA TAG ACT CGG GAG G | CCA ACC AAT GGG ACC GAA AGA TA |
