# Supplementary material for: Expression Patterns of Genes Involved in Sugar Metabolism and Accumulation during Apple Fruit Development
Source: PLoS One. 2012 Mar 7;7(3):e33055. doi: 10.1371/journal.pone.0033055 (PMC3296772; doi:10.1371/journal.pone.0033055)
Supplement: Table S8 — Linear regression equations between enzyme activity (y, µmol mg−1 Protein h−1) and the relative expression level (x) of its genes, y = ax+b, during apple fruit development. (DOC) [file pone.0033055.s008.doc]

**Table S8** Linear regression equations between enzyme activity (y, µmol mg^-1^ Protein h^-1^) and the relative expression level (x) of its genes, y = ax + b, during apple fruit development.

| Enzyme | Gene | a | b | r^2^ | ***p*** |
| --- | --- | --- | --- | --- | --- |
| SDH | *MdSDH1* | -0.103 | 3.238 | 0.756 | 0.13 |
|  | *MdSDH2-9* | 0.343 | -0.805 | 0.701 | 0.163 |
| CWINV | *MdCWINV2* | 0.086 | 0.404 | 0.994** | 0.003 |
|  | *MdCWINV3* | 0.081 | 0.446 | 0.980** | 0.01 |
| NINV | *MdNINV1* | 0.134 | 0.056 | 0.621 | 0.212 |
|  | *MdNINV2* | 0.177 | -0.256 | 0.946* | 0.027 |
|  | *MdNINV3* | 0.135 | 0.126 | 0.981** | 0.009 |
| vAINV | *MdvAINV1* | 0.028 | 0.781 | 0.941* | 0.030 |
|  | *MdvAINV2* | 0.027 | 0.793 | 0.903* | 0.050 |
| SUSY | *MdSUSY1* | -0.323 | 7.532 | 0.126 | 0.645 |
|  | *MdSUSY2* | 0.157 | 3.421 | 0.789 | 0.112 |
|  | *MdSUSY3* | 0.167 | 2.959 | 0.819 | 0.095 |
|  | *MdSUSY4* | 0.131 | 2.995 | 0.385 | 0.379 |
|  | *MdSUSY5* | 0.174 | 3.305 | 0.979* | 0.011 |
| FK | *MdFK1* | -0.097 | 3.890 | 0.244 | 0.506 |
|  | *MdFK2* | 0.407 | 1.033 | 0.981** | 0.010 |
|  | *MdFK3* | 0.764 | -2.734 | 0.931* | 0.035 |
|  | *MdFK4* | 0.519 | -0.093 | 0.990** | 0.005 |
| HK | *MdHK1* | 0.372 | 1.319 | 0.817 | 0.096 |
|  | *MdHK2* | 0.416 | 1.472 | 0.977* | 0.011 |
|  | *MdHK3* | 0.290 | 2.437 | 0.908* | 0.047 |
|  | *MdHK4* | 0.250 | 3.115 | 0.913* | 0.044 |
|  | *MdHK5* | -0.063 | 5.533 | 0.753 | 0.132 |
|  | *MdHK6* | -0.214 | 7.218 | 0.833 | 0.087 |
| SPS | *MdSPS1* | -0.566 | 12.86 | 0.257 | 0.692 |
|  | *MdSPS2* | 0.261 | 5.239 | 0.971* | 0.015 |
|  | *MdSPS3* | 0.929 | 0.297 | 0.793 | 0.110 |
|  | *MdSPS4* | 0.068 | 4.987 | 0.781 | 0.116 |
|  | *MdSPS5* | 0.009 | 7.460 | 0.987** | 0.007 |
|  | *MdSPS6* | 0.045 | 7.256 | 0.981** | 0.010 |

*significant at *P*≤0.05; ** significant at *P*≤0.01
